# HDLCA: hunger driven lion clustering algorithm, a novel energy efficient and scalable clustering approach for underwater wireless sensor nodes

**DOI:** 10.1038/s41598-025-18043-5

**Published:** 2025-09-26

**Authors:** Shyamsundar Raghu, T. Shankar

**Affiliations:** School of Electronics Engineering, Vellore Institute of Technology, Vellore, India

**Keywords:** Underwater wireless sensor networks, Meta heuristic clustering, Network topology, Routing, Engineering, Electrical and electronic engineering

## Abstract

Underwater Wireless Sensor Networks (UWSNs), a subset of traditional WSNs, face critical challenges due to their reliance on non-rechargeable, irreplaceable power sources, making energy-efficient communication essential. This paper proposes a novel meta-heuristic clustering-based routing protocol inspired by the hunger-driven hunting and territorial behaviour of lions, termed the Hunger Driven Lion Clustering Algorithm (HDLCA). Unlike other approaches, HDLCA directly maps lion behaviour to sensor node dynamics, enabling adaptive cluster head selection and efficient sub-cluster formation based on energy levels and node proximity. The algorithm is evaluated using key performance metrics including residual energy, dead node count per round, first and last node death, and throughput. Simulation results show that HDLCA optimizes these metrics effectively compared to EERBLC, EECMR, LEACH, and K-Means Clustering. Specifically, HDLCA achieves improvements in network longevity by 23.3%, 14.37%, 34.04%, and 59.91% when compared to EECMR, EERBLC, K-Means Clustering, and LEACH respectively. Additionally, HDLCA exhibits strong scalability, noise resilience, and consistent throughput, making it a robust and efficient solution for underwater deployments.

## Introduction

 UWSN is a networking infrastructure that combines the functions of sensing, processing, and communication. The sensor nodes are deployed underwater under various circumstances and are utilized by the UWSN. These sensors are used for collecting the data, which is serially connected to the processing unit, where the obtained data is fused and sent for transmission. The processing unit of these sensor nodes uses computational logic and stores the data for processing and manipulation^1^. The underwater sensors contain a transceiver, which is helpful in both transmitting and receiving the data. All these units are powered up by a central power unit that works based on the built-in battery that lasts from a few hours to months.

**Fig. 1:**
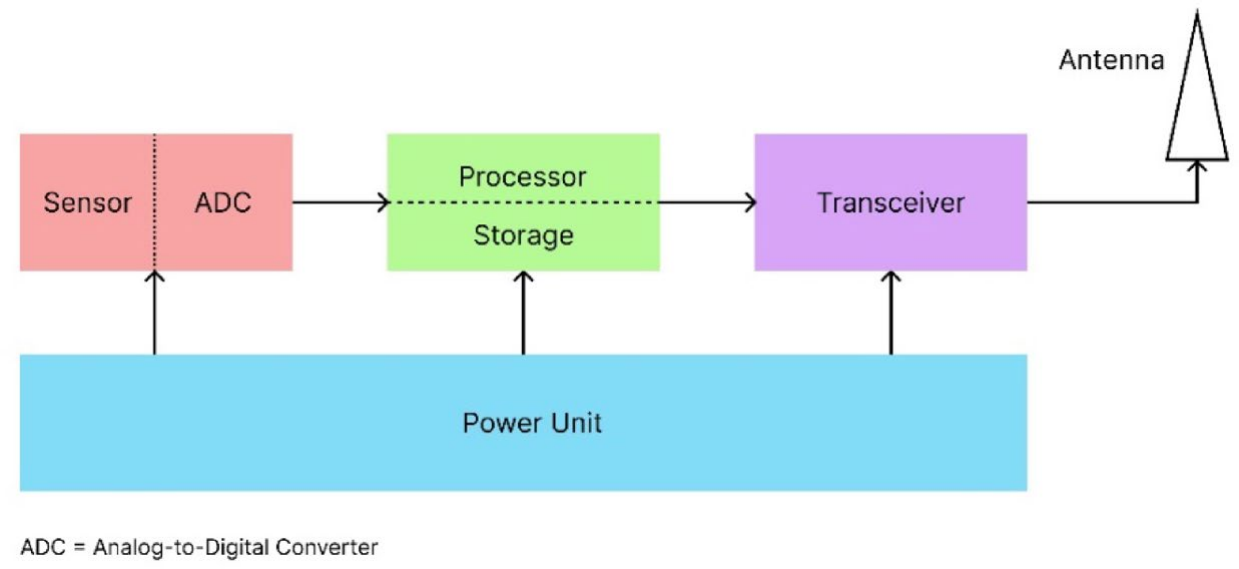
Hardware working unit of a sensor node.

Figure 1depicts the working model of a wireless sensor. The transceiver employed in an underwater sensor node typically uses an acoustic modem for communication. The deep-water channel is harsh and has a higher channel attenuation compared to the terrestrial environment. The channel mitigations this environment provides are accounted for by multiple factors such as path loss, noise, multipath fading, and Doppler spread. As mentioned earlier, the challenges have forced us to deploy these nodes with an acoustic modem rather than the conventional RF modem, as they are prone to higher attenuation underwater^2^. Acoustic communication underwater requires two main instruments, a hydrophone and a sonar. A hydrophone is a microphone that can be used underwater. It uses a piezoelectric transducer, which converts sound into electric signals. Sound Navigation and Ranging (SONAR) propagates the sound to navigate^3^.

### Motivation of the research

The deployment of UWSNs has become crucial for multiple applications like marine exploration, unmanned surveillance, defence systems, detection of toxic substances, etc. These applications often require a single loaded power supply lasting for months. Given that the channel is a high-level noisy channel, it is expected to have a minimum power loss of 5 dB. The frequent change in the channel condition constrains the network from adapting to a new topology. As mentioned earlier, the challenges have created a research scope among the researchers. The demand for energy efficiency motivates the development of an intricate and cognitive methodology that can be trained and applied for practical implementations.

The growing demand for higher efficiency has driven extensive research in hardware and software solutions. Hardware prevention strategies such as beamforming hydrophones, acoustic transducers, and AUV-assisted communications are utilised and have proven effective in mitigating channel impairments. Software innovations such as adaptive modulation schemes, adaptive equalization, and network layer solutions are also gaining popularity among researchers^4^.

This work introduces the Hunger Driven Lion’s Clustering Algorithm (HDLC) as a novel solution for the above-stated mitigation problems. The proposed algorithm is effective against higher noise environments and denser node deployments due to its transmission protocol and scalable property. The cognitive intelligence develops this algorithm to adapt to homogeneous and heterogeneous deployments.

The HDLCA surpasses EECMR, LEACH, K-means clustering, and EERBLC in longevity, throughput, and latency. Unlike the probabilistic clustering in LEACH, HDLCA appreciates real-time computational clustering based on each node’s fitness. The K-means clustering approach is centroid-based clustering, which can work well for static deployments, but must be trained for dynamic implementations. The HDLCA with single training can adapt to dynamic deployments.

 The rest of the paper is organised as follows: Sect. 2 briefs the type of protocols and Sect. 2.1 Describes the existing routing schemes and their mathematical models. Section 3 provides an introduction about the proposed HDLCA, Sect. 3.1 Explains the energy consumption model and network architecture. Section 3.2 describes the proposed Hunger Driven Lions’ Clustering Algorithm methodology and its mathematical models. Section 3.3 explains the transient phase, cluster head (CH) selection, CH capacity, and clustering strategy. Section 3.4 explains the CH’s steady phase routing ability and routing strategy. Section 4 describes the comparative results of the proposed algorithm with EERBLC, EECMR, K-means clustering, and LEACH performance. Section 5 provides the conclusion of the paper. The Table 1 describes the characters involved in the manuscript.

**Table 1:** Description of characters.

CHARACTERS	DESCRIPTION
UWSN	Underwater Wireless Sensor Networks
EECMR	Energy-Efficient Clustering Multi-Hop Routing
EERBLC	Energy Efficient Routing Protocol Based on Layers and Unequal Clusters
LEACH	Low-Energy Adaptive Clustering Hierarchy
a	Absorption coefficient
f	Operational frequency
A	Attenuation coefficient as a function of frequency and distance
d	Distance between the transmitter and the receiver
k	Spreading factor for an acoustic signal
N_t_, Ns, Nw, N_Th_	Turbulence noise, Shipping noise, Wave noise, Thermal noise respectively
P_0_	The minimum power required to establish a reliable communication
P_Transmission_	Transmission power required to attain a power level of P_0_ at the receiver end.
E_Transmission_	The energy required to transmit the data stream over a distance.
E_elec_	The energy required to transmit 1 bit of data
E_Reception_	The energy required by the receiver to receive the transmitted data stream.
P_reception_	The minimum power required by the receiver to receive bit of data.
E_Aggregation_	The energy required by the sensor to aggregate the data stream
E_DA0_	The energy required to fuse 1 bit of data.
Node_i_.Residual Energy	The residual energy of a sensor i at a particular round.
Node_i_.Initial Energy	The initial energy of all the deployed sensor nodes.
Node_i_.Order	The number of neighbouring nodes within the transmissible range of the Node i.
α, β (corresponding to the Eq. 12.)	The weights describing the influence of the parameter such that α + β = 1.
Hunger_i_	The parameter described to define the clustering capacity of the selected cluster head.

## Literature survey

The UWSNs work with limited computational processing and storage capacity. These nodes process complex signals at the receivers using higher-order filters. To overcome the challenges in Sect. 1, the signal strength of these sensor nodes must be greater than that of the terrestrial sensor nodes. The underwater sensor nodes are deployed in shallow and deep water, making replacing the battery difficult. The speed of propagation for an underwater acoustic wave is nearly. $\:1.5\times\:{10}^{3}$ m/s is heavily affected by several factors, such as salinity, depth, and temperature^5^. The acoustic channels of these UWSNs operate mostly below 30 kHz, which constrains their bandwidth. As the equation describes, with increasing frequency, there will be an increase in attenuation. (1)^6^:

1 $$\:a\left(f\right)=0.11\frac{{f}^{2}}{1+{f}^{2}}+44\frac{{f}^{2}}{4100+{f}^{2}}+2.75\times\:{10}^{-4}+0.003$$

Equation 1 estimates the absorption coefficient for a particular frequency and is calculated in dB/Km. As mentioned in Sect. 1.1The need for extensive research to increase the efficiency of the UWSN networks has driven many researchers to propose insightful ideas, and one among them is to improve the energy management through different routing strategies. The routing protocols are broadly classified into two categories based on how they forward data, which are multi-path and single-path routing protocols. In single path routing models, the sensor nodes transmit the data to the sink node directly, while the multi-path routing models utilise neighbouring nodes to transmit the data. The proposed methodology is categorised into multi-hop transmission protocols, adapting a clustering-based strategy for establishing an interconnection between the neighbouring nodes.

The clustering of nodes increases the network’s scalability and the network’s lifetime. By optimally clustering the nodes, the network can be trained to manage the traffic and balance the load of data aggregation. The clustering of nodes can detect the void space during the training and cluster the nodes in regions of higher nodal density. The results from the clustering approach adapted from natural phenomena have improved more than the traditional clustering approach^28^. Shankar et al. proposed an energy-efficient clustering based on the hybrid heuristic approach in wireless sensor networks^7^.Shyamsundar et al. proposed a cluster-based routing strategy for the UWSN using the Gravitational Search Algorithm, which increases the expectancy of the network by balancing the load and optimizing the routing paths^15^. Similarly, Panchal et al. has proposed an energy efficient clustering algorithm based on hierarchy and optimum grid-based head selection increasing the system efficiency significantly^8,27^.

### Exiting methodology

Over the years, various routing strategies have been introduced, and with regular research reviews, the clustering approach has gained popularity among researchers. This section discusses the existing methodologies employed in UWSNs adapting to cluster-based routing. The Table 2 compares different clustering algorithms based on the parameter for fitness evaluation, assisting nodes and efficiency.

**Table 2:** Comparison of state-of-the-art clustering algorithms in UWSN.

Protocols	Parameters	Assisting nodes	Efficiency	Throughput
EECMR^9^	Distance of sink from the node, Residual Energy, and head adopting time.	Relay Nodes	Medium	Moderate
	Distance of sink from the node, Residual Energy, Degree and the Depth Layer	Dual sink	Medium	Moderate
LEACH^11^	Head selection probability, present round	None	Low	Low
DBR^12^	Difference in depth, Packet reception time	Multi sink	Low	Low
EEDBR^13^	Depth and directional information	None	Medium	High
Levy Chaotic PSO algorithm^14^	Intra cluster distance, base station distance, cluster head energy, and member energy.	Node	High	High
GSA^15^	Distance of sink from the node, Residual Energy, Degree and the cubical coefficient	None	High	Moderate
HDLCA *proposed strategy*	Distance from sink, depth, degree nodes distribution	Nomad Lion Nodes	High	High

LEACH is a pioneer algorithm that clusters the nodes based on the probabilistic selection of cluster heads. The algorithm hierarchically organizes the clusters. Since the cluster heads spend most of the energy, the head is selected only when it belongs to the group of member nodes. Based on the history of the nodes, this group of members would compete to become the cluster head. Equation (2) gives the threshold value for the cluster head selection.

2 $$\:T\left(n\right)=\left\{\begin{array}{c}0,\:if\:n\notin\:G\\\:1-P\left(r\times\:mod\left(\frac{1}{P}\right)\right),\:if\:n\in\:G\end{array}\right.$$

Equation (2) explains the threshold for the competing nodes, where G denotes the group of member nodes in the last 1/P rounds and r denotes the current round.

Depth Based Routing^12^ is a geographic routing strategy that utilises a depth sensor to estimate the depth of the nodes to form clusters. The DBR takes advantage of multiple sinks and forms the routed network, without using a route table. The DBR is a greedy algorithm, which implies delivering the data packet to the sink node. The decreasing trend of the forwarding nodes would tend to avoid the void zones in the environment. DBR is resilient to the network topology but fails to be efficient as the sink nodes lose energy rapidly. The Energy Efficient Depth Based Routing^13^ is an advancement to the algorithm mentioned earlier, which considers energy in routing. The EEDBR compiles the depth and residual energy to estimate the forwarding node. The EEDBR optimises the energy consumption by prioritising the nodes with higher energy levels. Though the algorithm improves the efficiency of DBR, it still poses challenges like heavy computational load and lack of scalability.

The energy efficient algorithms such as EECMR^9^ and EERBLC^10^ increase the network’s lifetime significantly but fail to be reliable as the nodes are not confirmed to be clustered. The EECMR encourages the employment of relay nodes, which lie between the depth layers, that can be left alone despite having an optimal spatial position. While EERBLC encourages the nodes to be clustered based on competition time, the quickest nodes to respond are elected as the cluster heads. These cluster heads have an unequal cluster radius, which is determined based on the depth and energy level.

The LCPSO-CRP has advantages over existing algorithms because of its levy flight strategy and chaotic optimization. The LCPSO considers influential parameter such as base station distance, residual energy, cluster head energy and its member energy for optimization^14^. The GSA based algorithm has proven to be effective against harsh and dynamic environments but comprising with the complexity and processing load^15^. Communication is costlier with a higher population of sensory nodes, which is a trade-off between the delivery rate and data collection. This paper resolves the problems of load balancing, data coverage, void avoidance, scalability, and throughput.

## Proposed methodology

Underwater communication is always prone to attenuation and ISI for various reasons discussed in the Sect. 2. The loss produced by the medium due to channel mitigation would result in an attenuated signal. In contrast, a delay in bit synchronisation would result in ISI, which limits the Shannon’s capacity theorem. In addition, the speed of the sound varies due to the influence of shallow water conditions such as salinity, temperature, acidity, and pressure. Milica Stojanovic formulated a mathematical expression that accounts for the acoustic signal underwater to estimate the combined effect of all the above mitigation measures.

3 $$\:A\left(d,f\right)={d}^{k}\times\:a{\left(f\right)}^{d}$$

Equation (3) describes the attenuation estimated based on the distance *d* between the transceivers, the frequency of operation *f* which is responsible for the absorption coefficient as described in the Eq. (1) and the spreading factor *k.* Equation (3) Acoustic path loss can also be expressed in dB scale, which helps plot the graph for different frequency ranges and spatial distances.

4 $$\:0{log}\left(A\left(d,f\right)\right)=k\times\:10{log}\left(d\right)+d\times\:10{log}\left(a\left(f\right)\right)$$

Equation (4) describes the attenuation in dB scale.

**Fig. 2:**
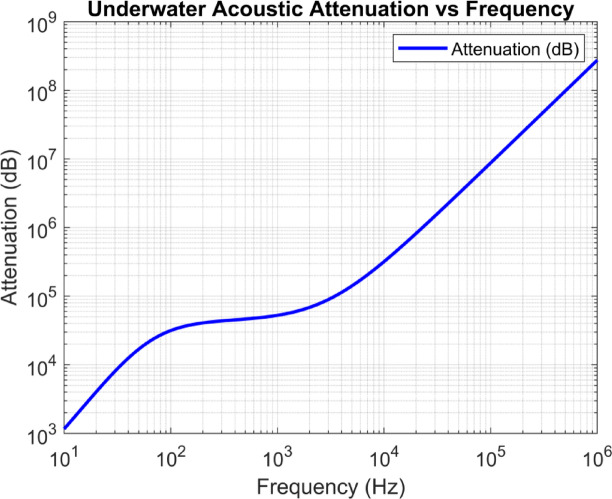
Increasing Attenuation with Operational Frequency.

Figure 2 demonstrates the effect of attenuation due to the increasing operational frequency. The generated plot was based on the formulation of Eq. (1) and Eq. (4). The graph clearly shows that choosing the right operating frequency is vital for transmission and energy conservation.

The important factor that contributes to the signal attenuation is ambient noise. Various natural sources produce this noise. The power spectral densities of these noises will vary based on the sources, such as the movement of water, wave currents, and pressure fluctuations. The surface wave generates the wave noise due to high-speed winds surfacing the water. The heavy movement of marine vessels produces the shipping noise. The shipping noise is a low-frequency acoustic noise that interferes with the transmission of the acoustic sensor signal. Due to the low frequency noise, shipping noise can travel long distances with minimal attenuation. The equations provide the empirical formulae of the power spectrum density of the turbulence, shipping, wave, and thermal noise. (5), (6),(7),(8) respectively^16^.

5 $$\:10{log}{N}_{t}\left(f\right)=17-30log\left(f\right)$$

6 $$\:10log{N}_{s}\left(f\right)=40+26{log}\left(f\right)+20\left(s-0.5\right)-60log(f+30)$$

7 $$\:10{log}{N}_{w}\left(f\right)=50+17{w}^{1/2}+20{log}\left(f\right)-40{log}(f+0.4)$$

8 $$\:\text{1,0}{log}{N}_{th}\left(f\right)=-15+20{log}\left(f\right)$$

Turbulence noise affects lower frequency ranges, while shipping noise impacts frequencies between 10 Hz and 100 Hz. The noise factor, denoted as ‘s’, is described by Eq. (6). Typically, the value of ‘s’ falls between 0 and 1. Additionally, thermal noise resulting from solar radiation and underwater activities is always the dominant factor for operating frequencies above 100 kHz.

### Energy consumption and network model

A sensor node must handle multiple functions that consume the reserved energy. These functions include sensing, processing, memory operations, transmission, reception, data aggregation, amplification, sleep, and wake-up mechanisms. This work scope lies in finding the appropriate transmission route through clustering, so this research considers only optimizing the communication spending, such as transmission energy, reception energy, and data aggregation. These factors are not absolute as they are interfered with by the environment, so we consider environmental factors such as path loss, multipath fading, and noise, whose mathematical formulations are quoted in the Sect. 3.

The power required by the transmitter to attain a power level of P_0_ at the receiver end is given by the Eq. (9).

9 $$\:{P}_{Transmission}={P}_{0}\times\:{d}^{2}\times\:{10}^{\frac{a\left(f\right)}{10}}$$

Equation (9) represents the required power level, while P_0_ is the minimum necessary to establish reliable communication between the transmitter and receiver. The term *d* denotes the distance between the transmitter and receiver, and *a(f)* represents the attenuation coefficient as prescribed by Eq. 1.

Equation 10 gives the energy required to transmit k bits of data over a distance d from the transmitter to the receiver.

10 $$\:{E}_{Transmission}=k\times\:({P}_{Transmission}+{E}_{elec})$$

Equation (10) represents the transmission energy. The term *k* represents the number of transmission bits, and E_elec_ represents the energy spent to transmit one bit of data. The term P_Transmission_ represents the power required to transmit the data, which is quoted in the Eq. (9). A sensor node requires considerable energy to receive the data; unlike transmission energy, reception energy is independent of the distance. The Equation gives the mathematical formula for the reception energy (11).

11 $$\:{E}_{Reception}=n\times\:k\times\:{P}_{Reception}$$

Equation (11) represents the reception energy a sensor node requires to receive *k* bits of data from *n* number of neighbouring nodes. The processed data by the sensor must be fused for a viable transmission and it is denoted by the Eq. (12).

12 $$\:{E}_{Aggregation}=k\times\:{E}_{DA0}$$

Equation (12) represents the aggregation energy required to fuse *k* bits of data, where E_DA0_ denotes the energy required to fuse one bit of data.

#### Network model

The proposed methodology adapts a static random deployment in the underwater environment. The sink is usually located at the deployment’s centroid position, and the results are derived based on these assumptions. The deployed environment is cubical of various dimensions. The network is treated with equal importance as concerned with the clustering phase, and the cluster heads are later grouped according to the depth for priority-based routing. The proposed algorithm has been tested under homogeneous and heterogeneous deployments, with non-uniform initial energies. The environment is shallow, and thus the value of *k* in the Eq. (4) It is taken to be 2 for cylindrical spreading. The operating frequency is assumed to be 30 kHz.

### Hunger driven lion clustering algorithm

This section introduces the Hunger Driven Lion Clustering Algorithm, a novel bio-inspired approach for energy efficient clustering in underwater wireless sensor networks. HDLCA is designed to mimic the hunger-driven behaviours of lions, where available resources dictate the decision making and movement, and its strict territorial bounding behaviour^17^. HDLCA effectively balances the network by handing off the load to the network, reducing computational overhead and increasing the network’s lifetime. The characteristics of the lion with respect to the clustering methodology have been discussed in the sub Sect. 3.2.1 and which explains adaptation from the lion’s behaviour to the clustering algorithm.

#### Hunger driven hunting

The noble character of a lion (Panthera Leo), who hunts only when hungry, helps them conserve energy and avoid unnecessary efforts. Usually, a lion’s pride would consist of a primary male who is dominant and decides about the portion of food each lion gets from the hunt based on the assistance^18^. The alphas are usually the group of hunting lioness that hunts the prey and bring it to the pride. The betas such as cubs and young lioness would assist the alphas in the hunt. Adapting the family architecture of the lions to the UWSN has a greater deal for resource management, leadership rotation, and scalability.

**Table 3:** Family architecture map to clustering in UWSN.

Role of the lion	UWSN Equivalent Role	Function
Dominative Male	Base station	The central authority decides the cluster heads and forms the link
Alpha – Hunting Lioness	Cluster heads	Forms a cluster for the significant population, collects data, aggregates the data, and transmits it.
Betas - Cubs and young lions	Assisting heads	It forms a cluster and acts as a cluster head for a smaller population.
Prey	Cluster Members	Senses data, aggregates it, transmits it to the cluster head, and returns to sleep.

The role of each node is described in the Table 3. The dominant male would decide the part of the meal for each of the members in the pride. The alpha would hunt and have superiority over the cubs and younger lions. Each alpha and beta would only participate in the meal that the dominant male ordered. This system efficiently managed the resources with a compromise between all the lions. Inspiring by this behaviour, a cluster-based approach for sensor nodes has been derived mathematically in the Sect. 3.3. The main features differentiating males, alpha and beta, are their greater potential for hunting and strength. In the case of UWSN, we assume each node is estimated based on its residual energy and order. Thus, the dominant male, the base station, systematically assigns its role in the family.

#### Territory marking behaviour of the lion

Lions are highly territorial animals that use vocalization and scent marking to establish their territory. Territory marking is essential for lions to maintain dominance and ensure access to the resources^19^. They are marking their territory either by urine marking or roaring, which can be heard from 8 km away. Lions actively patrol their territory to ensure that no intruders breach their boundaries. Some lions, known as nomads, live in solitude, constantly moving across without fixed territory. These lions are mature young lions who are forced out of their pride to establish their pride. These lions would avoid confrontations and live as scavengers for survival. These lions would eventually challenge the pride males to claim a pride^20^.

**Table 4:** Role of each in making the Territory.

Role of the lion	Role in UWSN	Function
Alpha	Cluster head	Forms the central cluster, just like an alpha taking over larger ground
Beta	Assisting heads	Forms the minor cluster with minimal ground to support alpha (Cluster head)
Nomad	Intermediate node	Formed an intermediate connection between the cluster heads for efficient transmission.

The role of each node in the deployment has been discussed in the Table 4. The proposed algorithm considers parameters of a working node, such as residual energy, and the count of neighbour nodes, which is called the order for establishing the architecture, assigning the role, and transmission route. A node is assigned as a cluster head, i.e., alpha head, when it has the highest residual energy in its neighbourhood and covers a vast population of nodes, i.e., pride. A node that might have a higher residual energy, but its prey population is vast, i.e., neighbour nodes, will then be assigned to assist the cluster head, i.e., beta, from its neighbour and form a minor cluster. The territorial behaviour of a lion would prevent any other lion from becoming an alpha. Thus, each cluster (pride) is assigned one cluster head (alpha or beta).

**Fig. 3:**
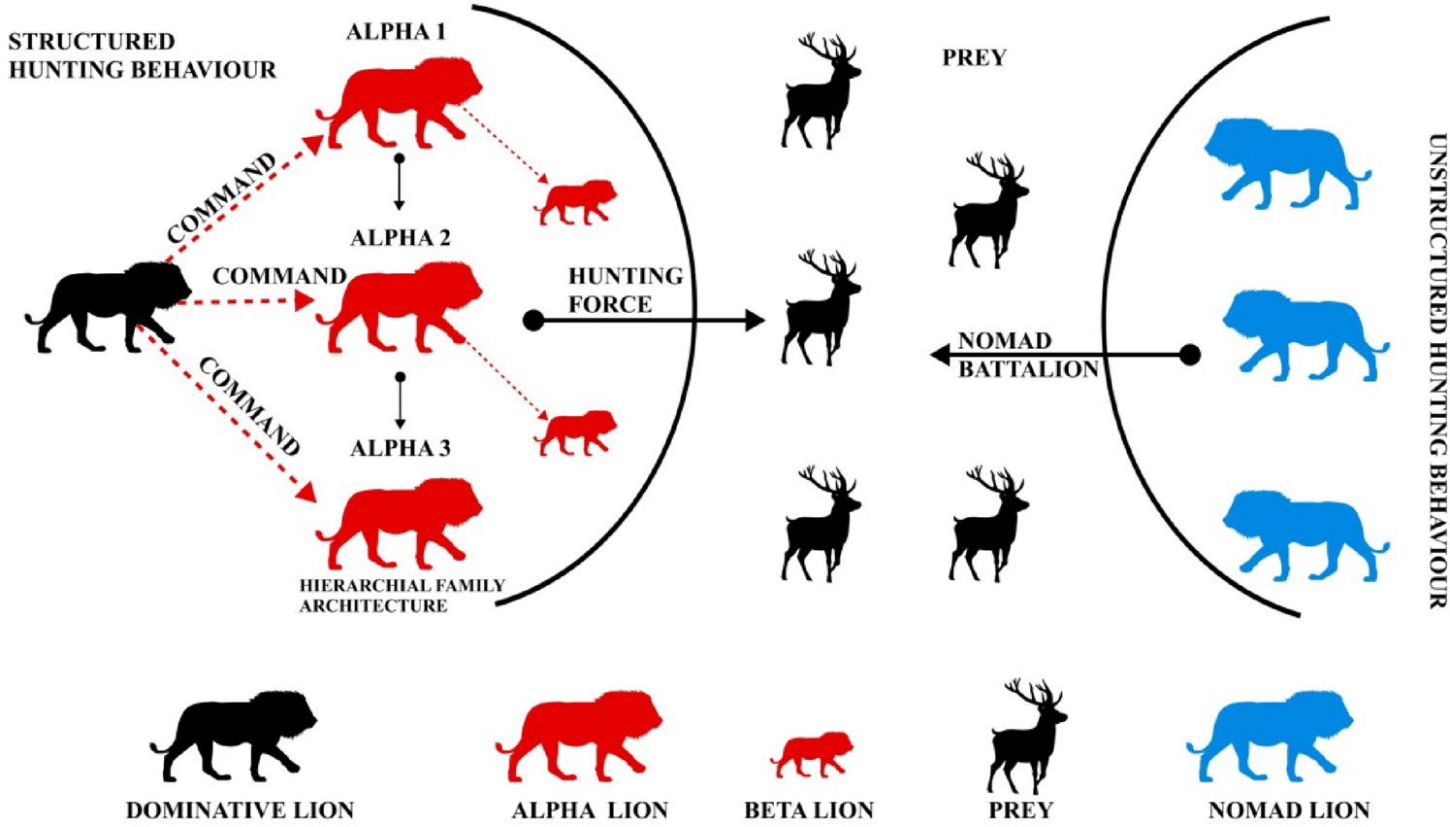
Lion’s Family Architecture showcasing the behaviour of hunting.

The Fig. 3 shows the hunting behaviour of the lion as described in the Sect. 3.2.1 and Sect. 3.2.2. The dominant lion commands the pride and manages the family architecture. Pride is hierarchically ordered based on strength, and the young lions are assigned to be betas. The hunting force approaches the prey while the betas help the alphas to hunt effectively. It is noticeable from the Fig. 3 the pride has a structured hunting behaviour, while the nomadic lion hunts with sheer strength.

In the proposed HDLCA framework, β nodes function as assisting cluster heads within the local pride (cluster), helping the alpha (main cluster head) in intra-cluster communication and data forwarding. In contrast, nomad nodes are dynamically selected intermediate nodes that do not belong to any fixed pride and are used to facilitate communication between geographically distant cluster heads, particularly in sparse underwater deployments where direct transmission is unreliable or energy intensive. These nomad nodes are selected based on their residual energy and link quality and are only activated when no reliable direct route exists. To prevent routing loops and redundant transmissions, a dynamic scheduling mechanism is integrated where a node’s participation as a nomad is periodically reassessed. Additionally, each cluster restricts the number of active forwarding paths, ensuring that nomads are sparsely and efficiently deployed. This mechanism not only minimizes resource wastage but also enhances network connectivity without compromising energy efficiency or significantly increasing latency.

#### Trophic ratio

The trophic ratio is the ratio of biomass or energy transfer between different layers of the food chain. A natural ecosystem possessing a prey-predator model would have a healthy trophic ratio. This trophic ratio sustains the biodiversity. Only 10% of the energy is transferred to the next level at each level^21^. The absence of an apex predator leads to overpopulation of secondary consumers, subsequently leading to vegetation depletion in the area. This can lead to a massive disturbance in biodiversity. The reverse effect would also bring a massive calamity in the ecosystem, as the elevated population of apex predators would decrease the secondary consumer population and subsequently lead to uncontrolled vegetation growth. Both imbalances can cause ecological instability, making it essential to maintain the trophic ratio. Adapting the concept of trophic ratio to the UWSN, it is crucial to keep the ratio between the cluster head to cluster members, as cluster heads consume significantly more energy than cluster members, ensuring efficient energy distribution and longevity.

### Clustering strategy

A deployed Underwater Sensor Network can have single-hop or multi-hop communication. The proposed methodology encourages the sensor nodes to form clusters among themselves to hold a transmission route through multi-hop communication. The cluster formed by these nodes would consist of a single central cluster head, to which the remaining nodes would transmit data in a timely and synchronized manner^22^. During the transient state, the sensor nodes would broadcast their spatial and depth information to one another. These data as a packet would then be transmitted to the base station to estimate the relative weight of each node. There are multiple existing clustering strategies, which are explained in the Sect. 2.1This research presents a novel clustering strategy inspired by lions’ hunting behaviour. A cluster would be formed through stages: information broadcast, cluster head selection, and cluster formation.

#### Information broadcast

At the initial stage of the transient state, each sensor node broadcasts a packet of data containing the depth and residual energy information, which are crucial for clustering and routing. Usually, this packet of data is 200 bits, but it varies indefinitely. Neighbour nodes would receive these packets, each with a timeout clock for receiving the packet. The receiver nodes whose clock reaches its timeout would aggregate the received data based on the time synchronization mode and forward their packet^23^. These nodes follow the TDMA mode of communication, where only a pair can communicate at a time, while utilizing the whole channel. The transmitted packet would have encrypted data about each node ID, depth, energy, and cluster history. The data packet arrives from the bottom nodes to the top and eventually reaches the base station. The base station decrypts the packet to get the insights and assigns the weights for each node. Based on the assigned node, the base station would form a cluster table, which is then transmitted to the nodes through CCHA (Compressed Cluster Head Advertisement).

#### Cluster head selection

A cluster head is the prominent cluster member and topologically at the cluster’s centre. The cluster head establishes the interconnectivity between the member nodes and the base station. The cluster head captivates the sensor nodes in its neighbourhood. The cluster head is selected based on the relative fitness of its neighbourhood. Each cluster head is elected based on the fitness during the initial information broadcast^24^. There are several selection strategies proposed based on different parameters, which are explained in the Table 2. The proposed methodology adapts the hunting behaviour of the lion. As discussed in the Sect. 3.2.1 A lion family structure has three main predators: the dominant lion, the alpha, and the beta. The dominant lion is the base station that handles all the heavier computations. The election between the alpha and beta happens after differentiating between the prey and the alphas, as the beta plays the subordinate role.

13 $$\:Fitness=\alpha\:\times\:\frac{{Node}_{i}.Residual\:Energy}{{Node}_{i}.Intial\:Energy}+\beta\:\times\:\frac{\:{Node}_{i}.Order}{Total\:EquationNumber\:of\:nodes\:},\:\alpha\:+\beta\:=1$$

The Eq. (13) explains the fitness of a node based on its residual energy, $\:{\text{N}\text{o}\text{d}\text{e}}_{\text{i}}.\text{R}\text{e}\text{s}\text{i}\text{d}\text{u}\text{a}\text{l}\:\text{E}\text{n}\text{e}\text{r}\text{g}\text{y}$ and its neighbouring population, $\:{\text{N}\text{o}\text{d}\text{e}}_{\text{i}}.\text{O}\text{r}\text{d}\text{e}\text{r}$. The nodes having a higher order are provisionally elected as heads, and based on the relative energy level, they are selected as alpha cluster heads. Each of these elected alphas has its clustering capacity, which can be metaphorically compared to the hierarchy of the lion, and it is calculated relatively. The concept of hunger can be understood in terms of a lion’s family hierarchy; that is, a lion possessing superiority in terms of strength gets to have a significant part of the prey, while the lion at the lower level shares a little portion of the meal. Likewise, an alpha node with higher residual energy will be superior to others. This method introduces a term called hunger, which defines the clustering ability of a node based on the residual energy.

In Eq. (13), the parameters α and β are weighting factors that balance the influence of residual energy and node order (which reflects the local node density or proximity). For our simulations, α and β were initially set to 0.7 and 0.3, respectively, based on empirical tuning to ensure energy-aware cluster head selection without overwhelming the network with high-density clustering. However, these parameters are not static. They are dynamically adjusted at the beginning of each round based on the standard deviation of node energy levels. For example, when the energy variation across nodes increases (i.e., more heterogeneity), a higher α value is used to prioritize energy. Conversely, in a more uniform energy scenario, β is increased slightly to promote even spatial distribution. This dynamic adaptation allows the HDLCA to respond effectively to changing network conditions, maintaining clustering efficiency and prolonging network lifetime.

14 $$\:{Hunger\:}_{i}=\frac{{Node}_{i}.Residual\:Energy}{Average\:Cummulative\:energy}\times\:Number\:of\:active\:nodes\:$$

15 $$\:Average\:Cummulative\:Energy=\frac{\sum\:_{i=1}^{Number\:of\:acvitve\:nodes}Residual\:energy}{Number\:of\:active\:nodes}$$

Equations (14) and (15) describe a node’s clustering ability and the average cumulative energy, respectively. The described equations are proven to be scalable as they are causal and do not depend on static values. This system of equations can be applied to any micro- to macro-level network. A significant part of a network that consumes energy is the cluster heads, and it is essential to elect the cluster heads optimally. As described in the Sect. 3.2.3The trophic ratio is crucial to sustaining biodiversity; hence, it is also important to maintain it in UWSN. Given that it maintains the trophic ratio, the election of beta nodes substantially happens after the election of adequate alphas. It is empirically found that 15% of cluster heads in the total population return maximum diversity and longevity. The trophic ratio can be used to tune the network’s scalability.

The election of beta nodes is crucial to support the alpha nodes in governing the local territory, in case the elected alpha nodes cannot captivate the whole neighbouring population. For example, based on the Eq. (14). An alpha has the capacity to capture six prey nodes, but its neighbourhood has ten nodes. It can only cluster the best six, which are nearer, and the rest will be left alone. To avoid this problem, we elect a beta node to cover the rest of the nodes and complete the cluster. Although the beta node has its own cluster, it is only to assist the alpha node and has a higher chance of forming a single route between them.

The “Hunger” value introduced in Eq. (14) quantitatively expresses the clustering capacity of a node. A node with higher residual energy compared to the network’s average cumulative energy experiences a higher hunger value, metaphorically representing its dominance and ability to lead a cluster. The hunger metric directly influences the cluster formation hierarchy: nodes with higher hunger are considered for alpha roles, while others take on beta or follower roles. This ensures that cluster heads are selected based not only on absolute energy but on relative energy superiority, thereby improving energy balance across the network.


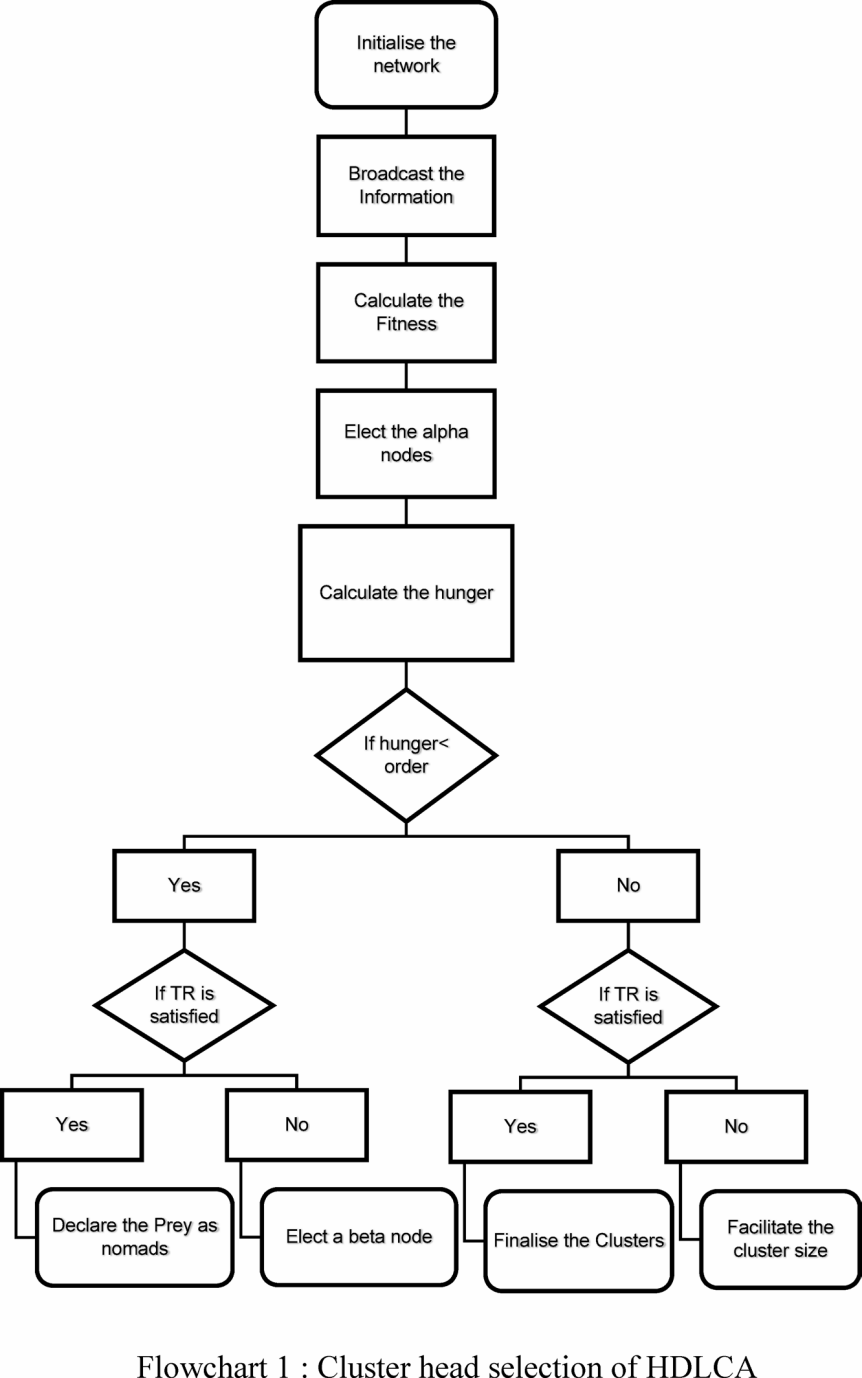


#### Cluster formation

The formation of a cluster is solely based on the spatial orientation of the alpha and beta nodes and the relative distance between them. The elected heads, an alpha or a beta, would mark their boundary like a lion marking its territory. The marked boundary can shape a sphere around the cluster head. Each cluster head would send a JOIN message to its neighbouring nodes based on the timeout clock. This JOIN message would contain depth, relative distance, TDMA slot, and hunger level information. If the receiving nodes receive a JOIN message from different heads, they will choose the head with the highest residual energy, the local alpha node. If the alpha node has captivated the nodes to reach its hunger, it will assign the beta node as a cluster head and captivate the rest of the nodes. Upon accepting the offer, each member would send a RESPONSE message to confirm its membership in the cluster.

### Routing strategy

The steady state establishment has a crucial role in the longevity of the UWSN network. The strong underwater currents can rapidly shift the sensor nodes from their positions. The routing strategy must be reliable to overcome this issue by a quick response^25^. The proposed methodology utilises the setbacks from the HDLCA to form a reliable interconnection. As discussed in the Sect. 3.3.2. An alpha with higher hunger can cluster more prey nodes, while a lesser hunger can take the assistance of a beta node. While forming a routing path, it is always preferable to establish the connection between relatively closed nodes rather than farther nodes. By this concept, the betas, which are positioned close to the alphas, form the establishment with one another.

Each transmission requires a forward node, specifically an alpha node, which is positioned at a shallower depth than the transmitting node. The two main factors determining the forwarder are depth and hunger level. A node with a lesser order but a higher hunger level can be an effective forwarder, as its hunger can be satisfied through forwarding. The nomad nodes, which were taken out of the cluster because of the incapability of the alpha, can serve as the intermediate nodes. The nodes deployed in void spaces are also the nomadic lion, which can participate in inter-transmission. The alphas would send a message ROUTE to the base station containing the number of members’ depth and hunger information. The base station would then estimate the best route pair based on the relative distance and depth level, and the intermediate nodes are assigned to assist the transmission^26^.

The boundary marking behaviour inspired by the lion’s territory marking behaviour helps to bind the clusters and form a definite interconnection. The proposed routing approach increases the network’s reliability and efficiency. The proposed approach encourages the nodes to maintain proper synchronization as the established connection is highly adaptable due to the existence of nomadic nodes. The proposed methodology encourages multi-hop transmission by introducing nomadic and beta nodes closer to the alpha nodes.

**Figure:**
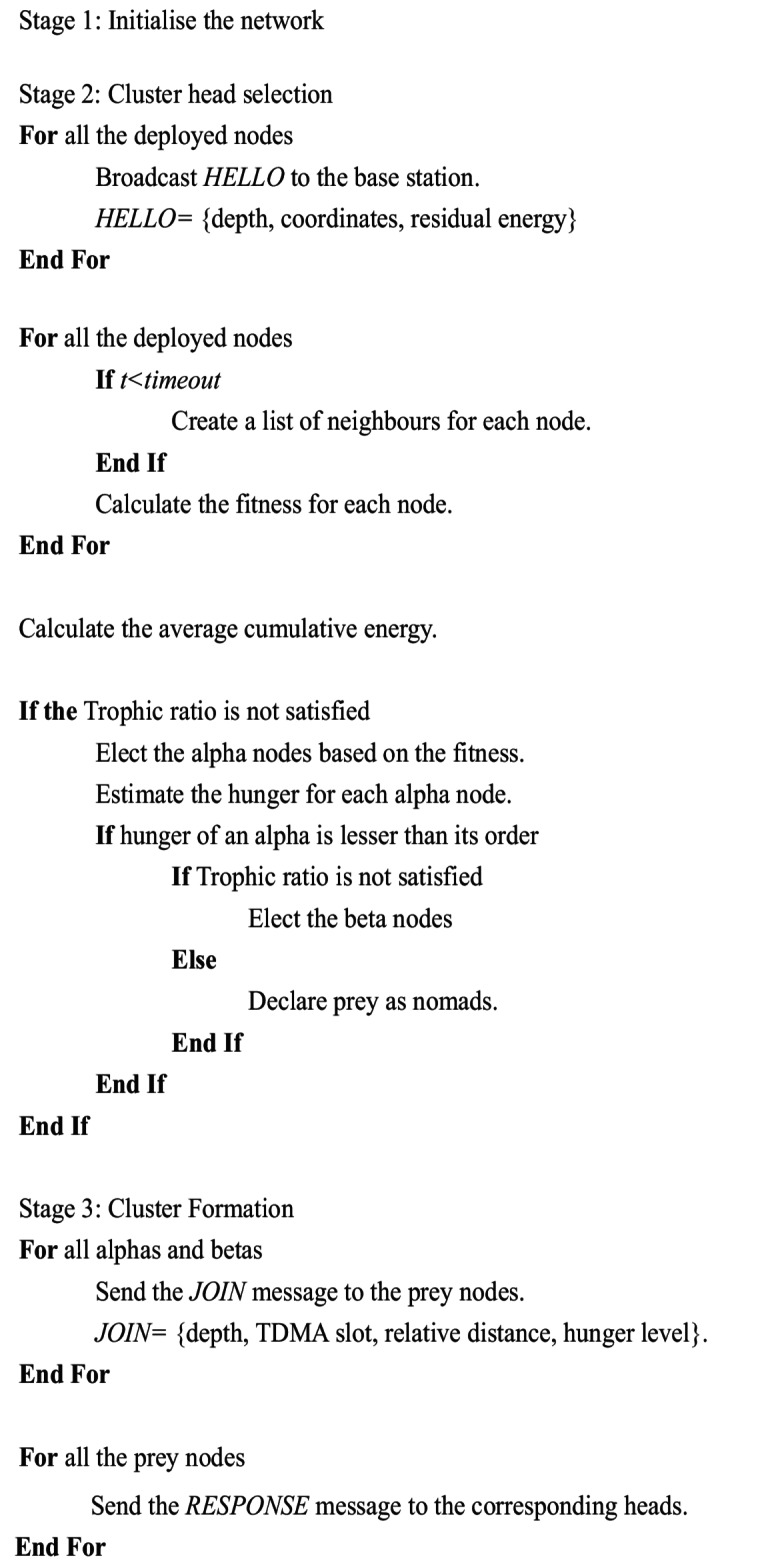
Algorithm 1. Hunger Driven Lion Clustering Algorithm

**Figure:**
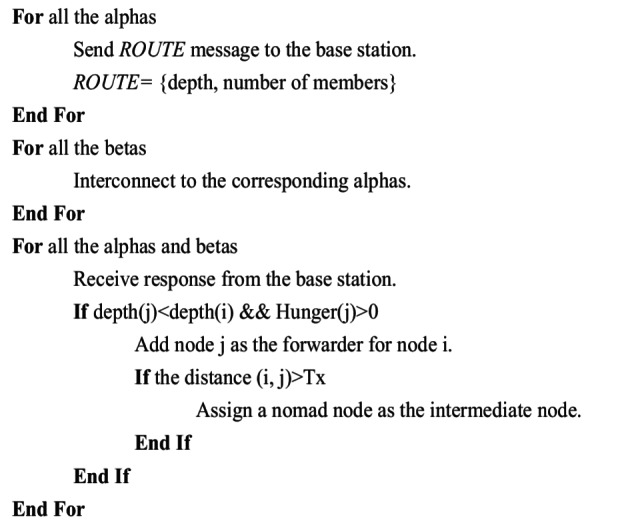
Algorithm 2. Routing Strategy for HDLCA

## Results and discussion

To validate the effectiveness of the proposed Hunger Driven Lion Clustering Algorithm (HDLCA), comprehensive simulations were conducted using MATLAB 2024b. All experiments were performed under consistent and controlled conditions to ensure fair comparison. The HDLCA algorithm was benchmarked against four well-known clustering techniques: LEACH, K-Means Clustering, EECMR, and EERBLC. Each algorithm was evaluated across identical network configurations, including area size, initial energy, data packet size, and transmission parameters. The simulations were conducted under both homogeneous (equal energy) and heterogeneous (random energy) node deployments. Additional scenarios incorporated varying node densities, cluster sizes, and both noisy and noiseless environments to test the adaptability, scalability, and robustness of each method.

**Fig. 4:**
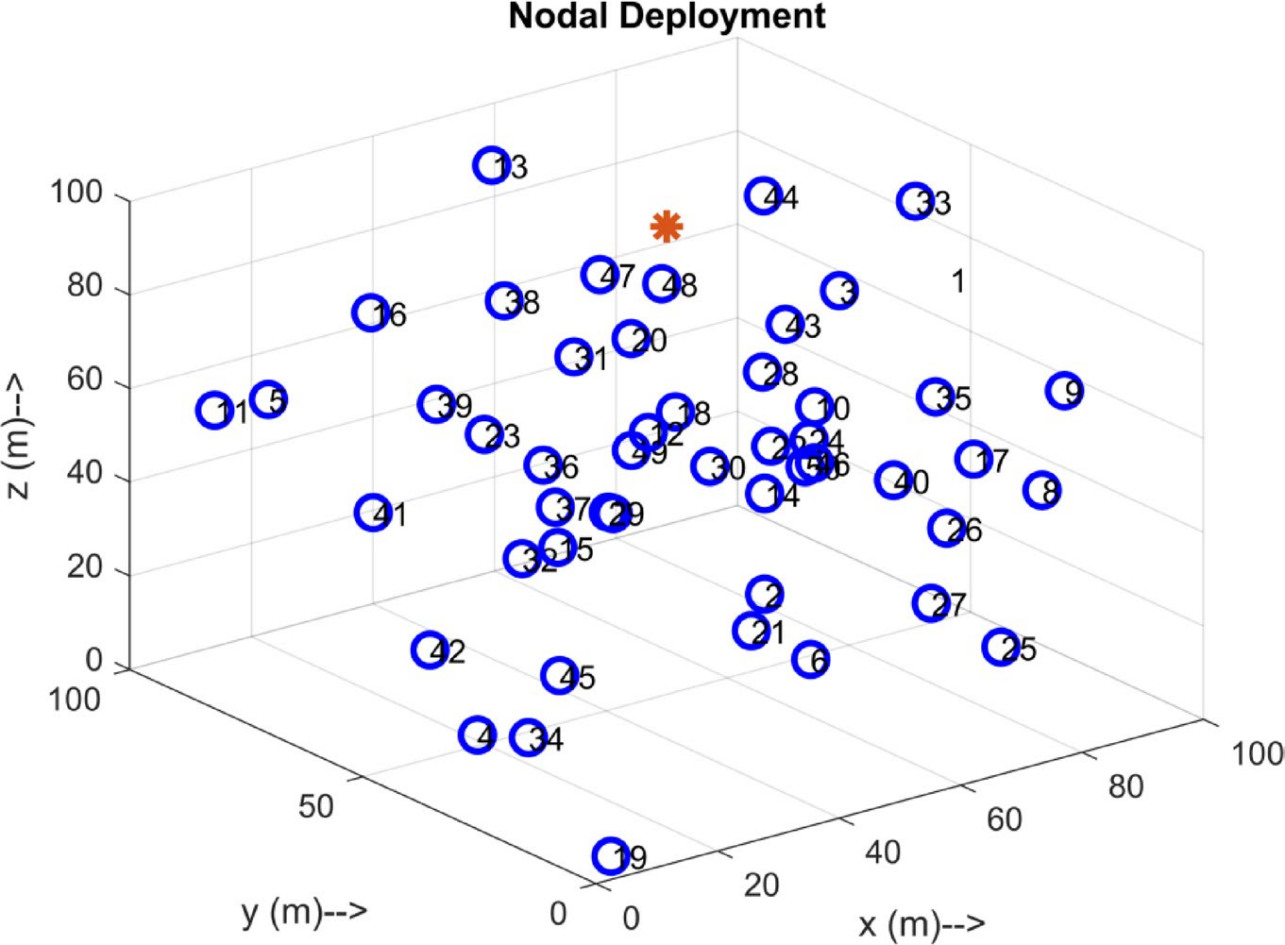
Deployment of 50 Sensor Nodes.

**Fig. 5:**
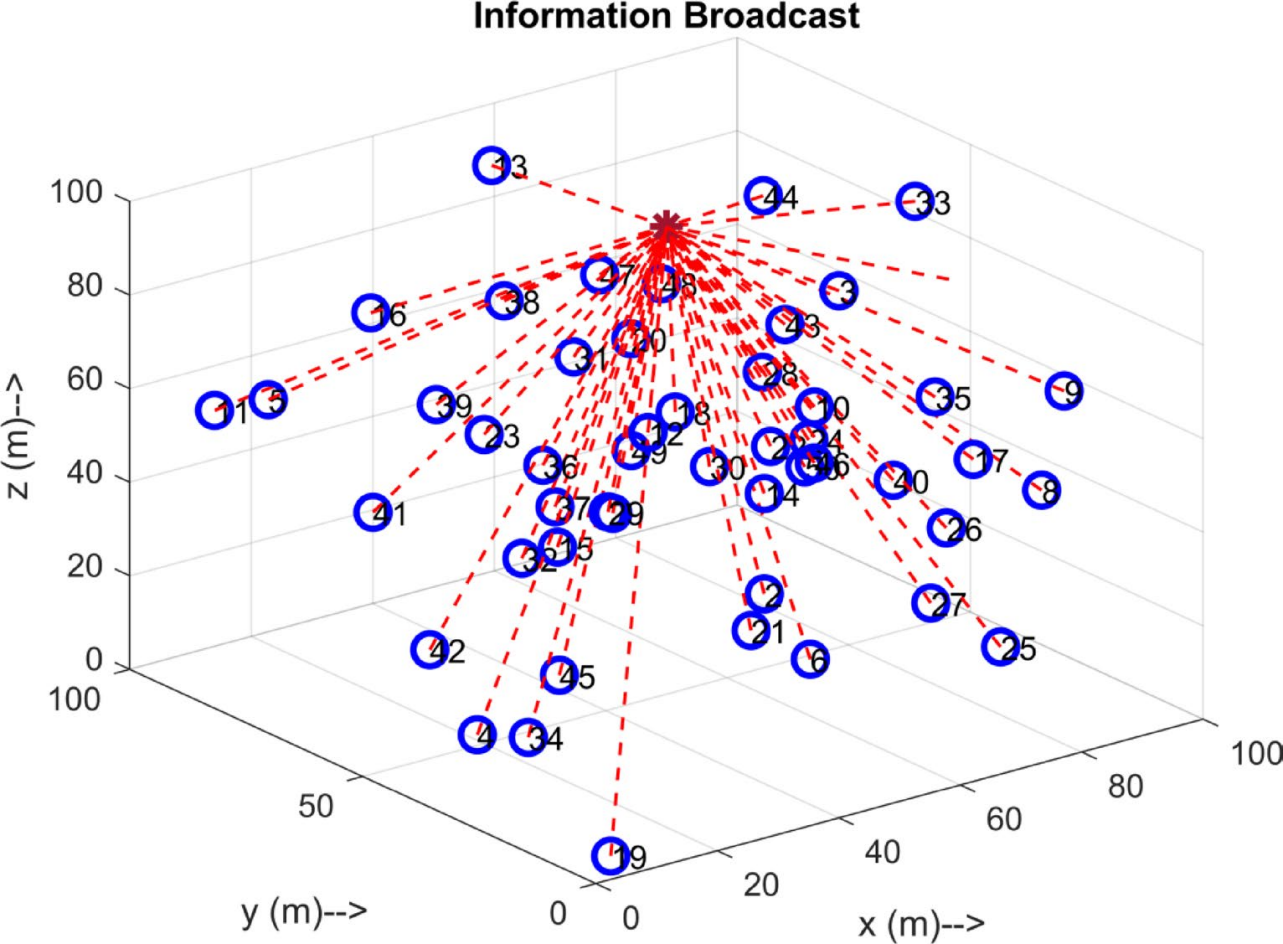
Information Broadcast.

**Fig. 6:**
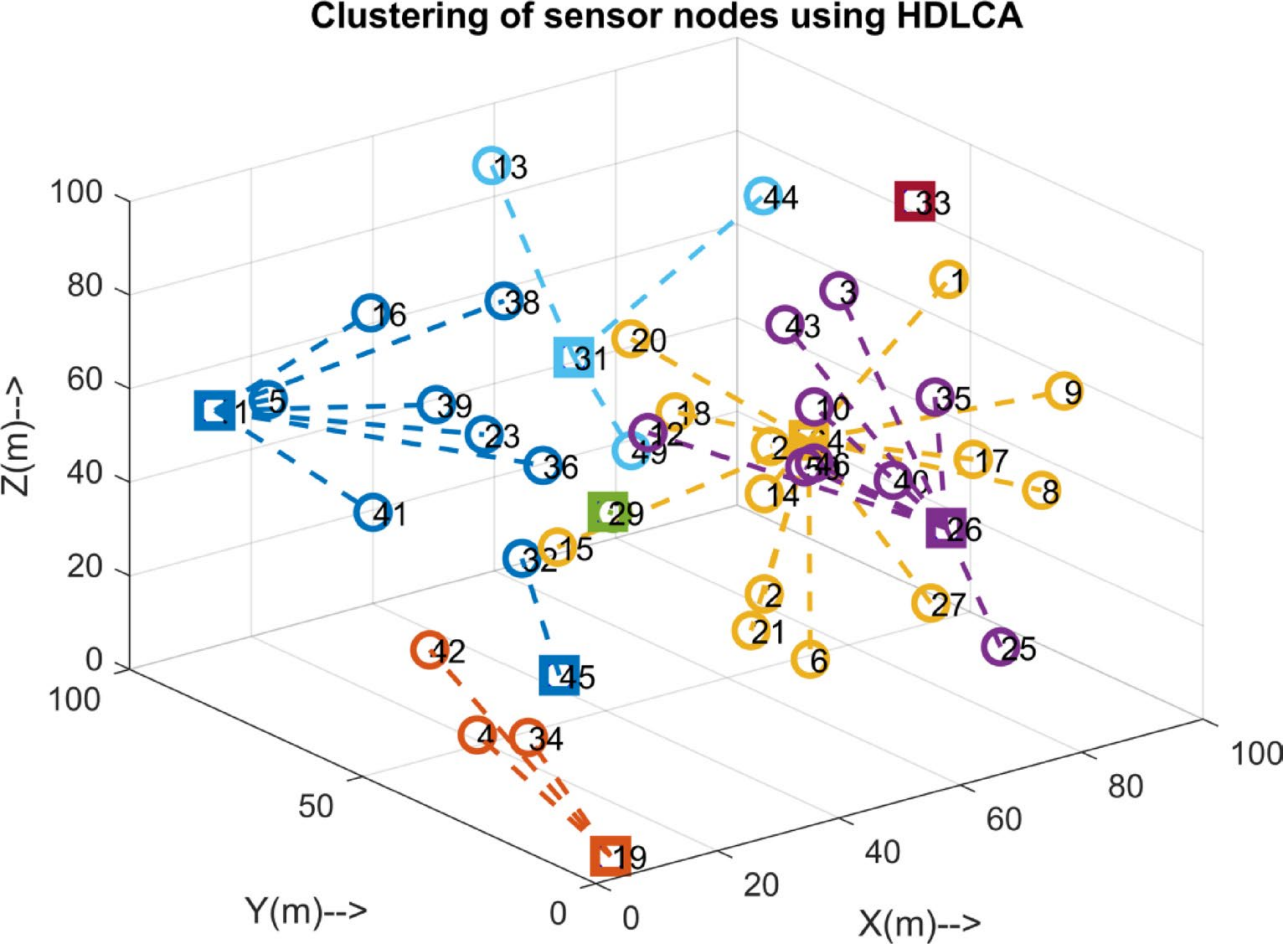
Clustering of 50 sensor nodes implemented using HDLCA.

**Fig. 7:**
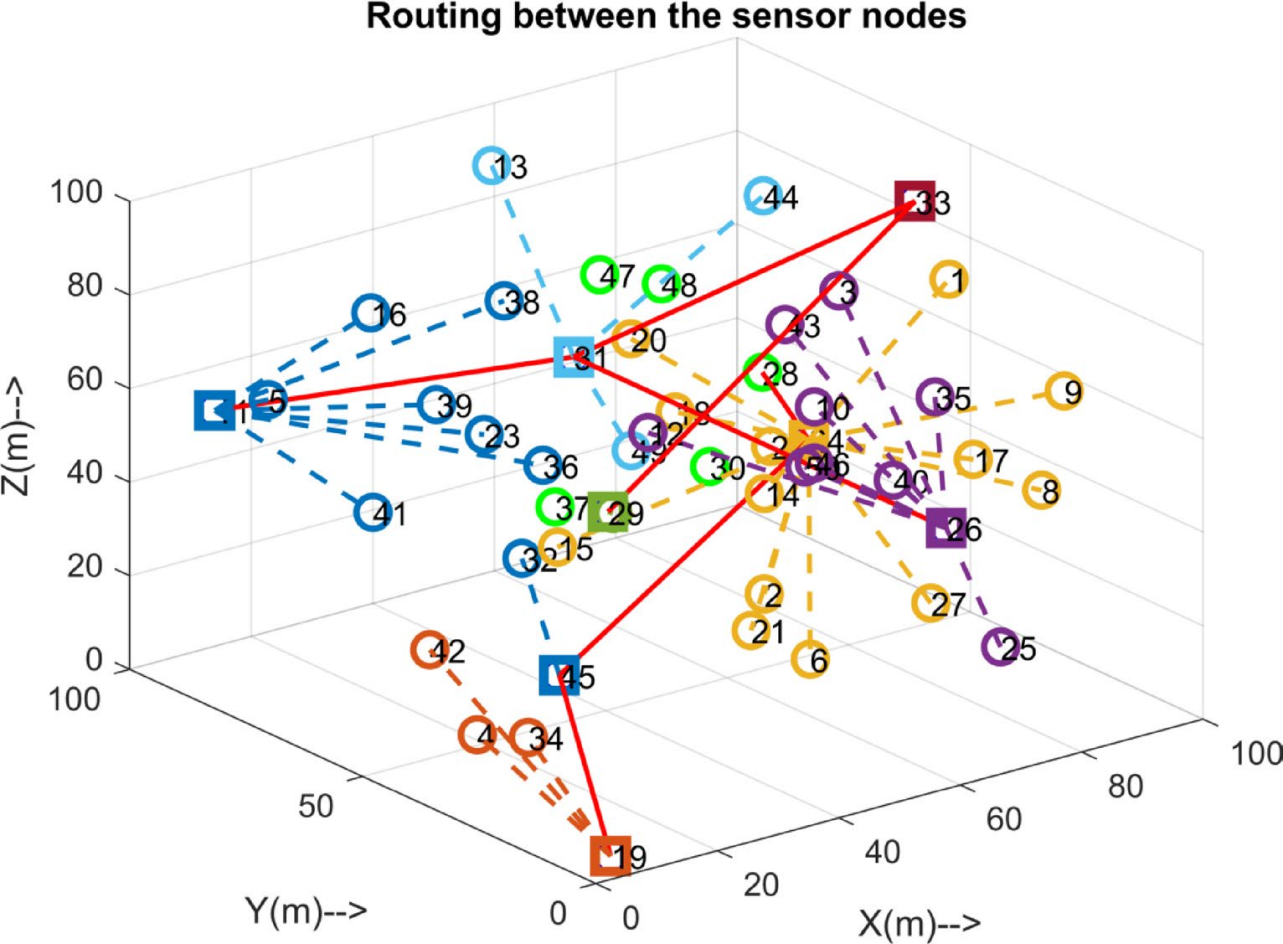
Routing of 50 sensor nodes.

**Fig. 8:**
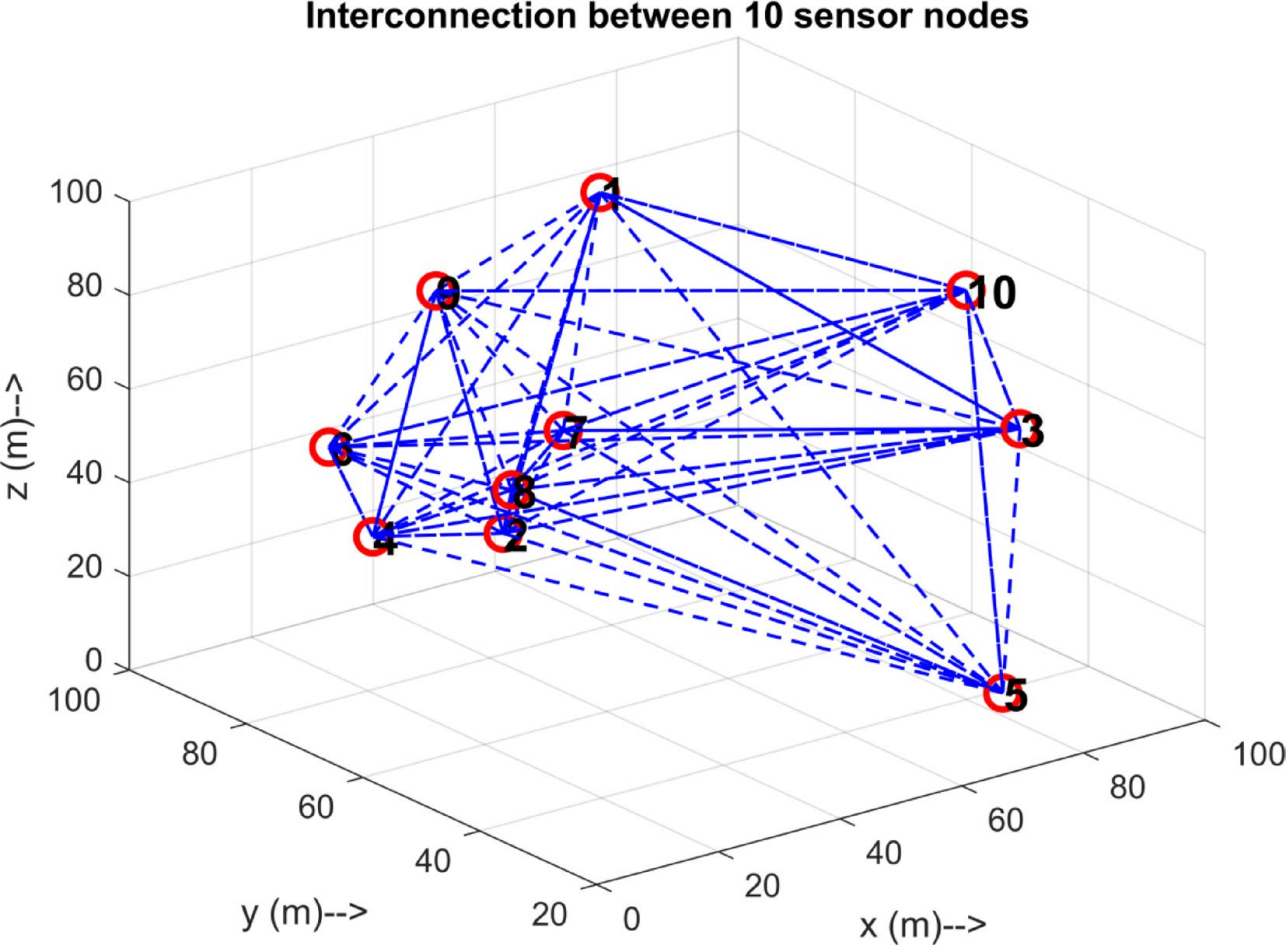
Interconnection between 10 sensor nodes.

The Figs. 4, 5 and 6, and 7 Illustrates the evolution of the interconnection through the HDLCA. The Fig. 4 depicts the random deployment of 50 sensor nodes across the underwater environment of 100 m x 100 m x 100 m in a homogeneous energy initialization. The sink of the environment is placed at the centre of the environment at a depth of 100 m. The Fig. 5 depicts the interconnection from each of the sensor nodes to the sink. The data packet sent from these sensor nodes contains the depth information, clustering history, and residual energy. These received data packets are aggregated at the sink and tabulated for clustering. The Fig. 6 illustrates the clustering strategy adopted from the HDLCA, and the following Algorithm 1. It is evident from the Fig. 6 The order level of the cluster head determines each cluster. An alpha whose hunger is lower than its hunger level reaches for an assisting cluster. The Fig. 7 demonstrates the routing mechanism of the HDLCA following the Algorithm 2. The beta nodes are initially left alone, and during the steady state phase, these beta nodes are interconnected to the alphas. The alpha nodes with lower depth are the end nodes that estimate their nearest neighbour for.

**Table 5:** Network setup Parameter.

Parameters	Value
**Network Area**	$\:500\varvec{m}\:\times\:500\varvec{m}\:\times\:500\varvec{m}$
**Number of Nodes**	100–300
**Data packet size**	200 bits
**Transmission Distance**	150 m
**Transmission Frequency**	30 KHz
**Initial Energy**	5 Joules
**Received power constant**	$\:5\times\:{10}^{-10}$ Joules
**Aggregation energy constant**	$\:5\times\:{10}^{-9}$ Joules
**Bit transmission energy constant**	$\:5\times\:{10}^{-9}$ Joules

setting up the connection. The alpha node with intermediate depths is a relay node connecting the lower and higher depth nodes. The Fig. 7 depicts the allotment of nomad nodes, which helps establish an interconnection between the alphas deployed in the void areas. Figure 8 illustrates an example of an interconnection involving 10 deployed nodes, each communicating with the others through distinct links.

### Homogeneous deployment

In this environment, 150 nodes were deployed initially. All the deployed nodes have the same energy of five joules. The proposed algorithm has been compared with state-of-the-art algorithms such as EECMR, EERBLC, LEACH, and K Means Clustering. To evaluate the performance of these algorithms, metrics like residual energy, number of dead nodes, and Transmission energy have been calculated. The network setup parameters have been listed in the Table 5.

**Fig. 9:**
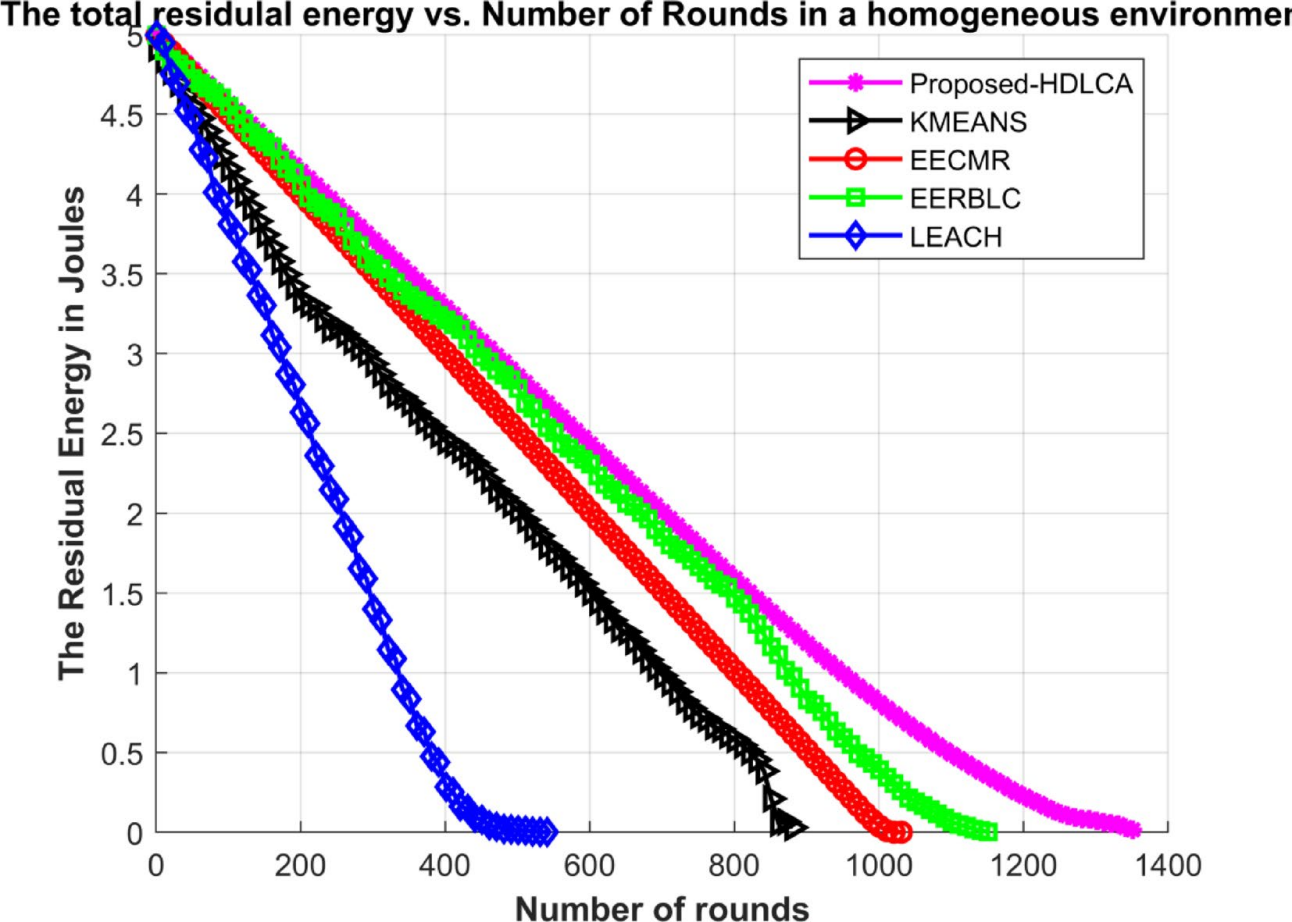
Average residual energy Vs. Number of rounds in a homogeneous environment.

**Fig. 10:**
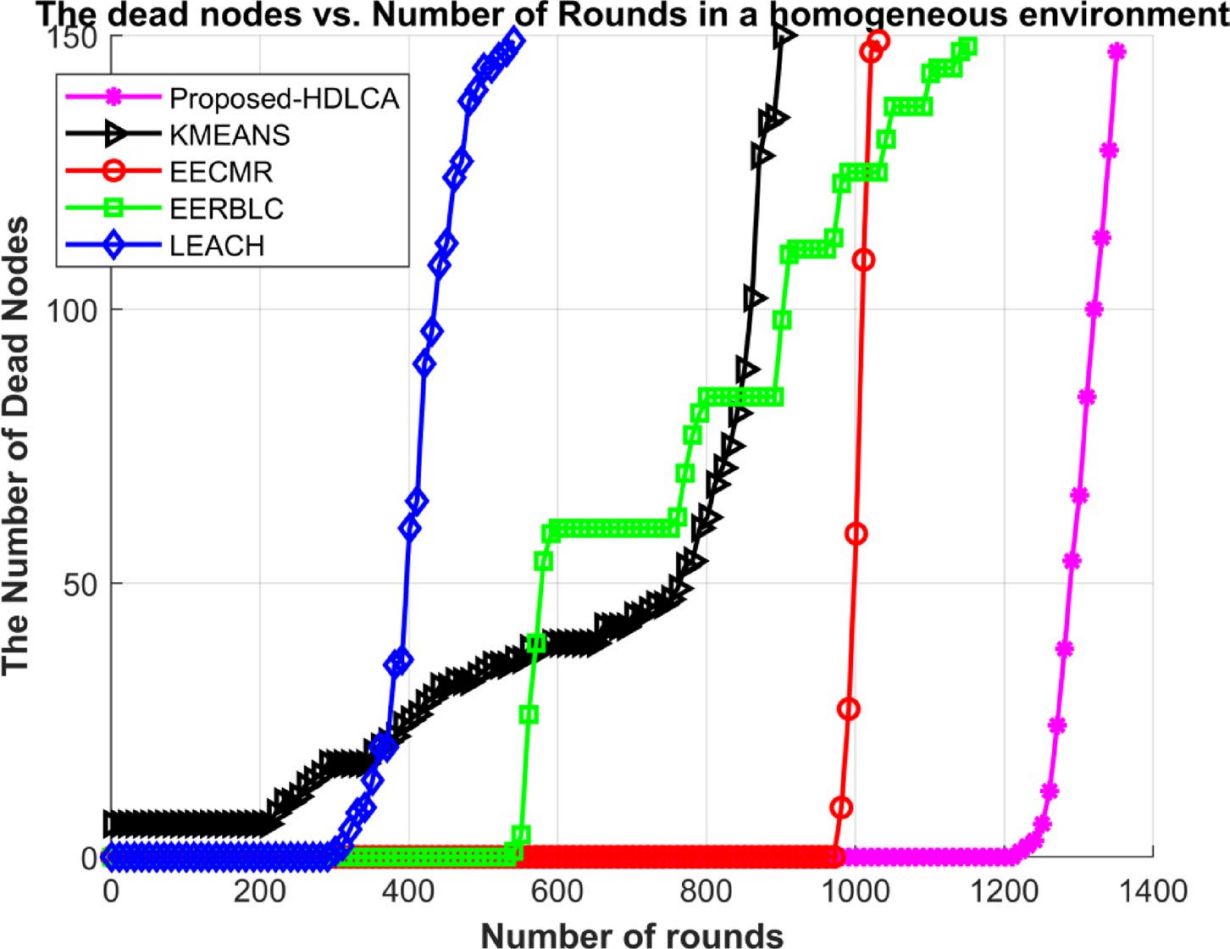
Number of dead nodes Vs. Number of rounds.

**Fig. 11:**
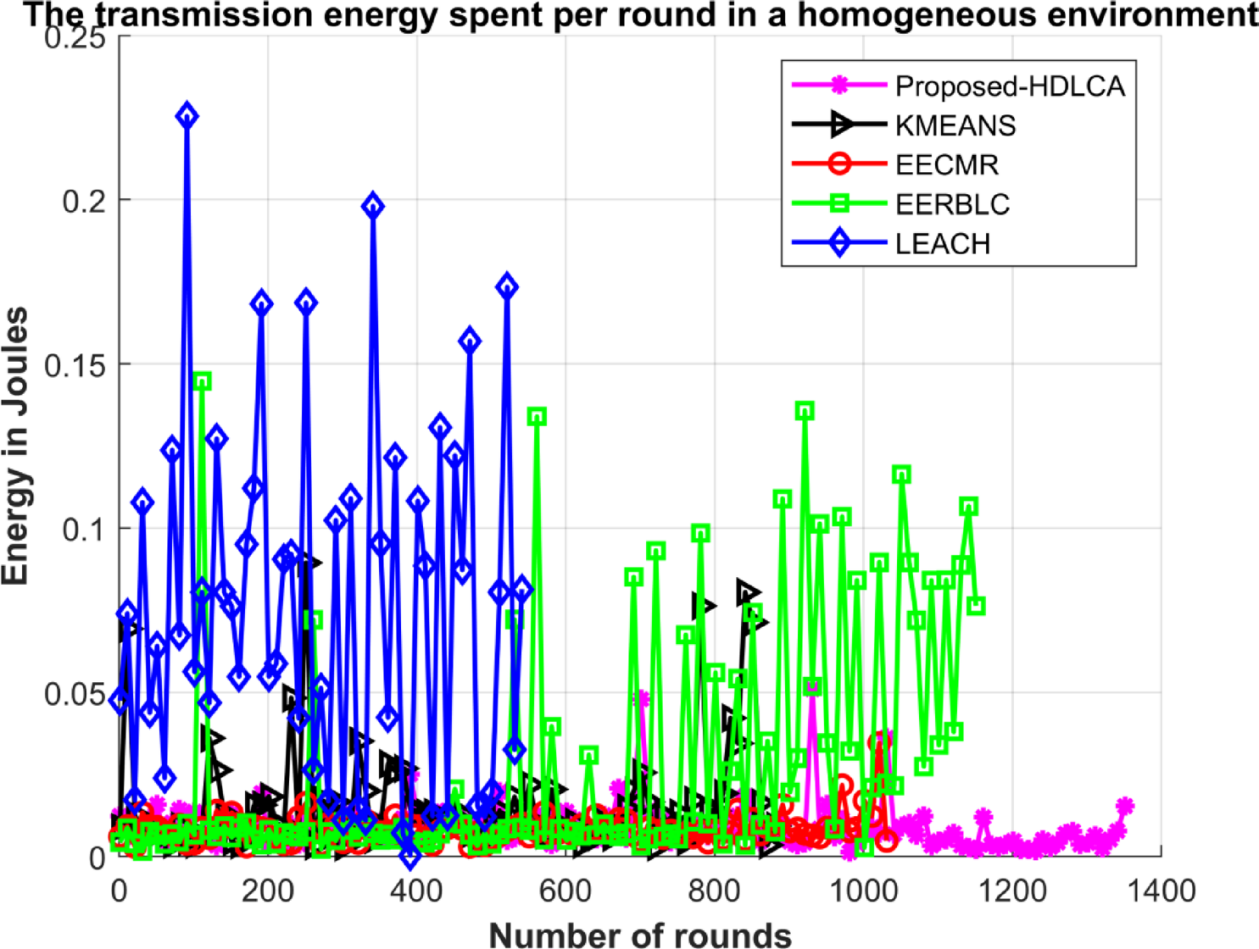
Transmission energy spent per round.

The Fig. 9 depicts the average residual energy spent with the growing number of rounds. The proposed methodology attains the maximum efficiency for the proposed setup. The performance of these algorithms can be explained by novel purpose of development. The EECMR algorithm which prioritises energy efficiency demonstrates a linear exploitation and higher expectancy in denser deployments. The EERBLC which prioritises energy efficiency and load balancing demonstrates stepped exponential exploitation.

**Table 6:** Expectancy performance of the algorithms in a homogeneous environment.

Algorithms	Number of Rounds	First Node death	Last node death	Rate of exploitation
Proposed- HDLCA	1357	1213	1357	Linear
EERBLC	1157	550	1157	Exponential
EECMR	1038	978	1038	Vertically peak
K Means Clustering	895	213	895	Higher order Exponential
LEACH	544	318	544	Intermediate

The Table 6 explains about the expectancy of the deployed sensor nodes. The EECMR shows a sharp spike in its expectancy due to its inability to balance the load. The Fig. 10 shows a rapid depletion of resources due to its inability to scale at lower order. The EERBLC implies an unequal clustering, and its effect can be seen through the Fig. 10, as the curve of exploitation shows a stepped response. The algorithm has a higher order load balancing, but it does not adapt to the environment quickly. This would affect the count of data packet reaching the base station due to quick loss of resource. The proposed HDLCA algorithm showing a superiority over the other state-of-the-art algorithms in terms of efficiency, throughput and load balancing. The main motive of proposing HDLCA is to be highly energy efficient and to balance the load. The Fig. 9 depicts the steady rate of energy consumption because of its load balancing capacity. The sub clustering system proves to be helpful in increasing the scalability of the network. It is evident from the Fig. 10, that among all other algorithms, the proposed HDLCA is having higher drive, of over 120 rounds after its first node death at 1213 rounds.

The EERBLC shows an improvement in its performance by 10% compared to EECMR. Despite of its expectancy, it is not resourceful because of low data acquisition at higher order. The pioneering algorithms such as LEACH and K Means clustering showing lower performance compared to the other algorithms. The K Means clustering exhibiting lower expectancy and lower drive. The algorithm also proves to be less resourceful because of the lower drive. The LEACH depicting the least performance in terms of efficiency and throughput. The performance of LEACH is lower because of its probabilistic election of cluster heads. The Fig. 11 demonstrates the transmission spent per round, the trend of the proposed HDLCA lies low and consistent compared to all the other competitive algorithms.

**Fig. 12:**
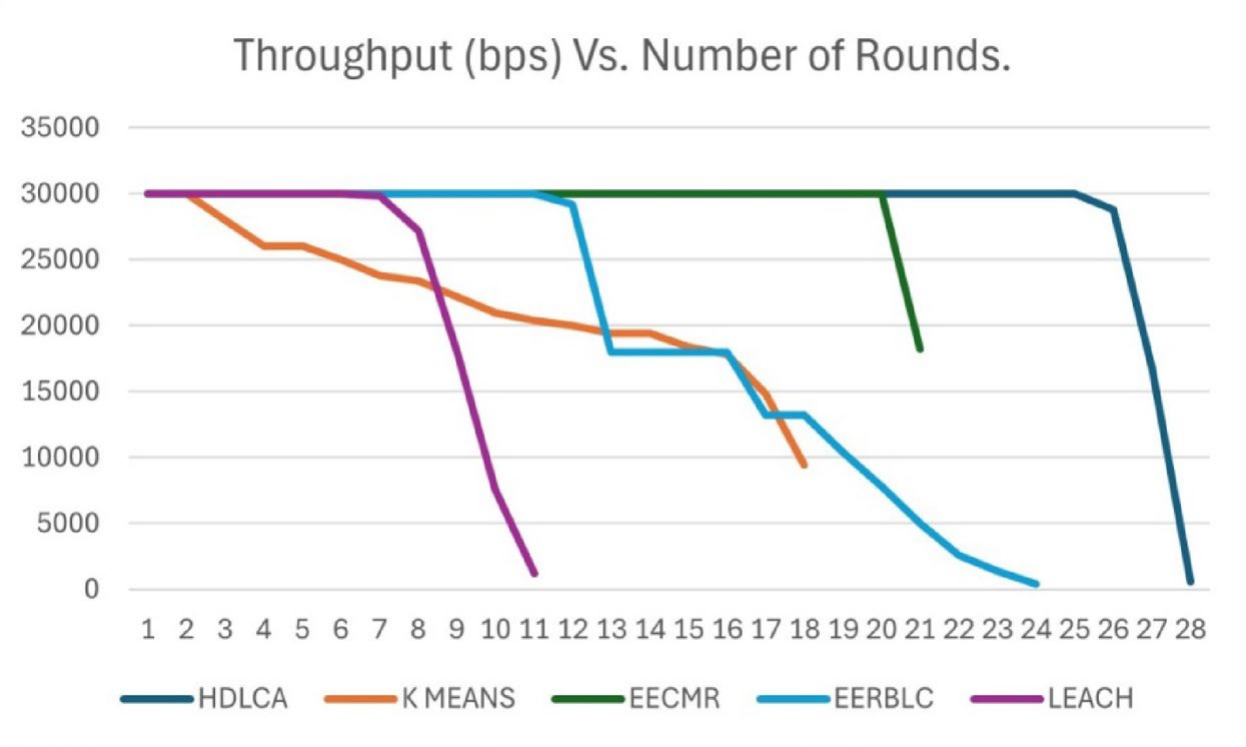
Throughput Vs. Number of rounds.

The acceleration of these algorithm has a significant effect on the data rate, since it is important for the network to keep constant throughput for reliable data transmission. The Fig. 12 depicts the throughput sampled at fifty rounds for better plotting. The Fig. 12 signifies the vitality of throughput over the longevity. The EERBLC showing better performance compared to the rest of the algorithms have a diminishing throughput starting from 600th round. The cause of this diminishing effect can be accounted because of the unequal clustering, that leads to void space. The EECMR shows better performance in terms of throughput compared to the EERBLC. The K Means clustering algorithm shows poor performance because of its lower order clustering heads. The proposed method, HDLCA shows superior performance compared to all the other algorithms because of its high adaptability to the environment. The improved performance can be attributed to the sub-clustering system implemented through beta nodes.

#### Number of rounds vs. Clustering radius

**Fig. 13:**
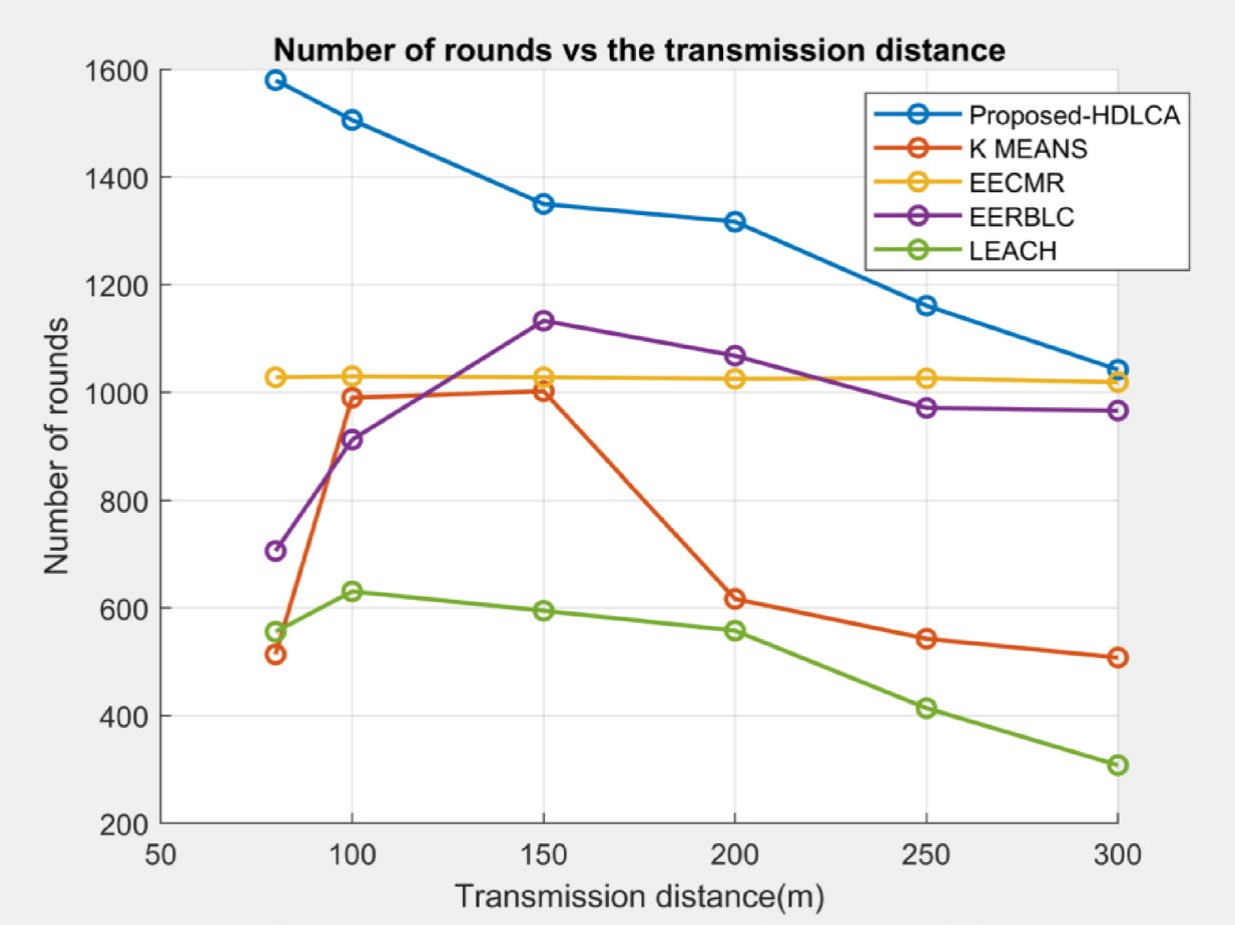
Number of Rounds Vs. Clustering Radius.

The Fig. 13 represents the longevity of the algorithms versus the clustering radius. The clustering radius plays a major role in energy consumption, since the transmission distances significantly tunes the consumption curve as defined by the Eqs. (3) and (9). The power consumption is squared proportional to the transmission distance. The performance of these algorithms can be explained by their clustering strategy. The performance of LEACH is highly influenced by the probabilistic selection of cluster heads which indeed affects the nominal clustering. The K Means clustering being a learning-based strategy clusters the nodes based on centroid and nominates the node nearer to the centroid as the head. The EERBLC exhibits unequal clustering throughout the layers from the seabed which helps the network to easily adapt the environment. The EECMR shows a constant performance throughout, because the algorithm was designed to maintain the throughput. The proposed methodology, HDLCA shows the best performance compared to all the other algorithms in terms of longevity. The cause of its performance can be understood from the sub clustering mechanism of the network. The alpha nodes nominate its beta nodes to maintain the population. The concept of trophic ration which was introduced in the Sect. 3.2.3, helps to maintain the throughput and gets adapted to the environment easily.

#### Longevity vs. Node deployment

**Fig. 14:**
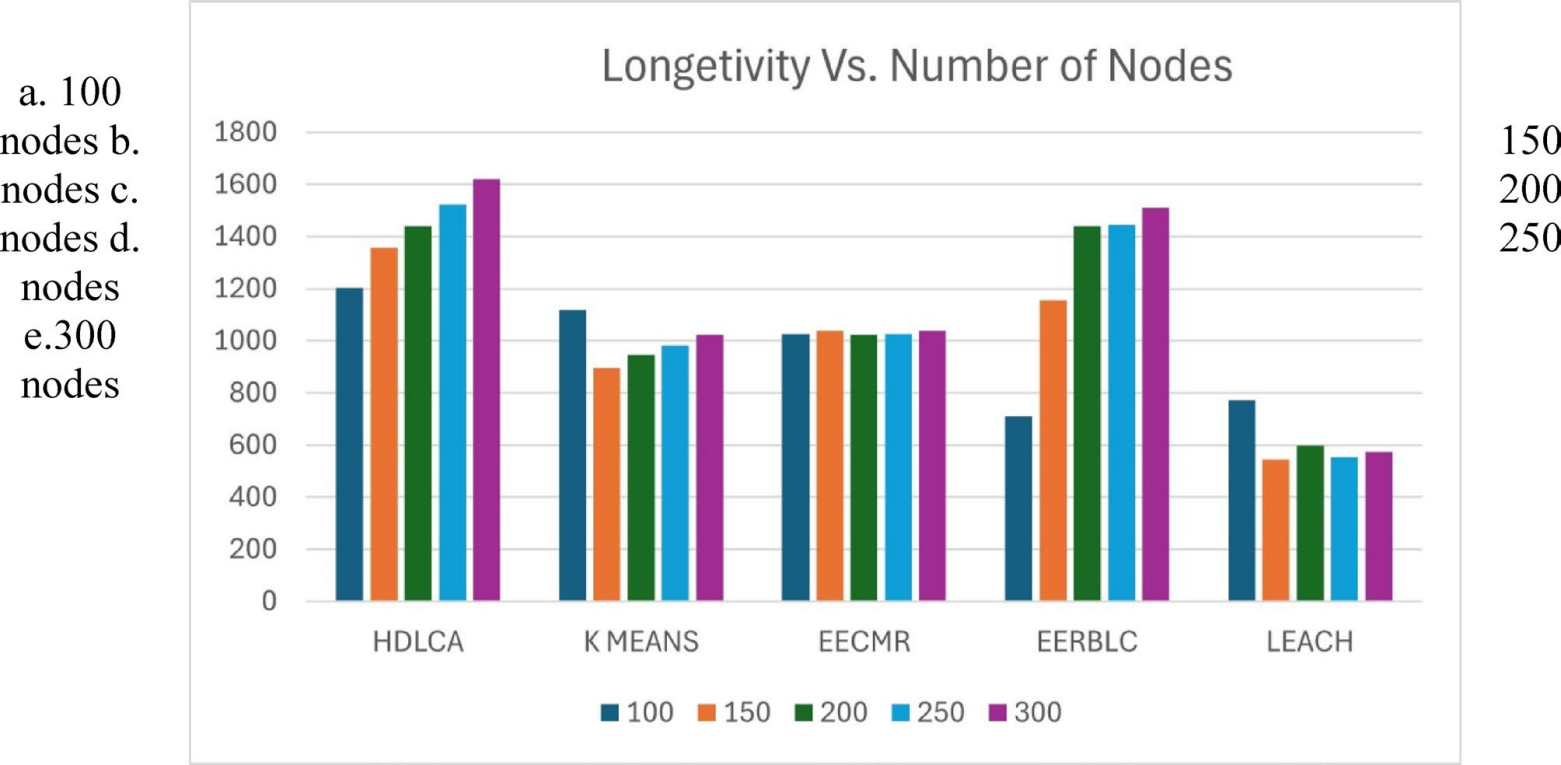
Number of Rounds lasted Vs. Number of Nodes deployed. (a) 100 nodes (b) 150 nodes (c) 200 nodes (d) 250 nodes e.300 nodes.

The Fig. 14 depicts the number of rounds lasted for different nodes deployment. The varying nodal deployment has been undertaken to test the scalability of the algorithms. The proposed methodology shows the highest scalability because of its sub clustering mechanism, which dynamically changes its clustering capacity to maximize the efficiency. The EERBLC giving competitive results as it was designed to be adaptable by clustering the nodes unequally. The EECMR shows constant performance which was previously reported in the Sect. 4.1.1. The motive of EECMR is to provide a constant throughput and it experimentally proven through Fig. 14. The underwhelming performance of the LEACH and K Means clustering can be understood from its clustering strategy.

### Heterogeneous deployment

In this environment, 150 nodes were deployed initially. All the deployed nodes have random initial energy. The proposed algorithm has been compared with the state-of-the-art algorithms such as EECMR, EERBLC, LEACH and K Means Clustering. To evaluate the performance of these algorithms’ metrics like residual energy, number of dead nodes, transmission energy has been calculated.

**Fig. 15:**
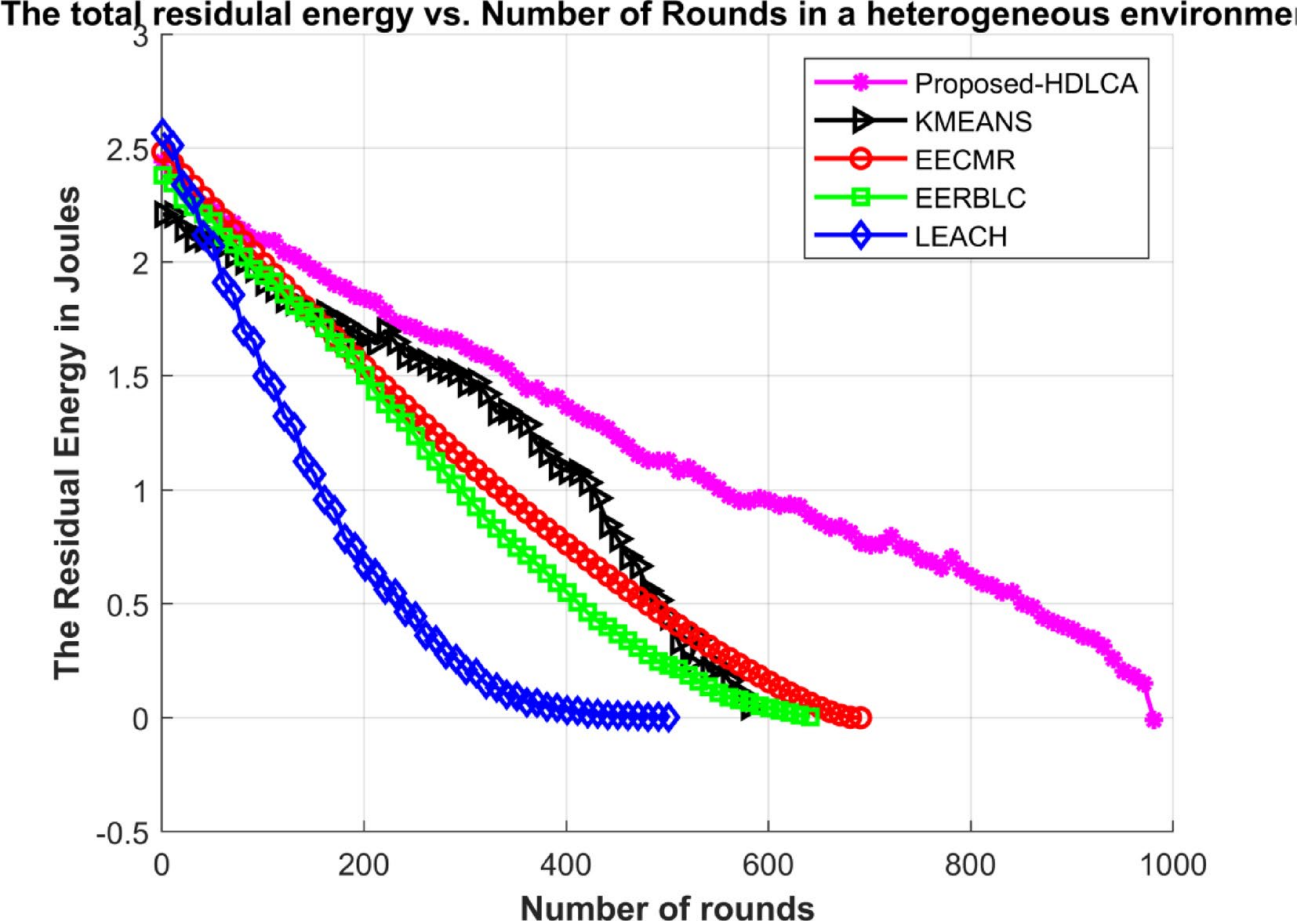
Number of dead nodes Vs. Number of Rounds in a heterogeneous environment.

**Fig. 16:**
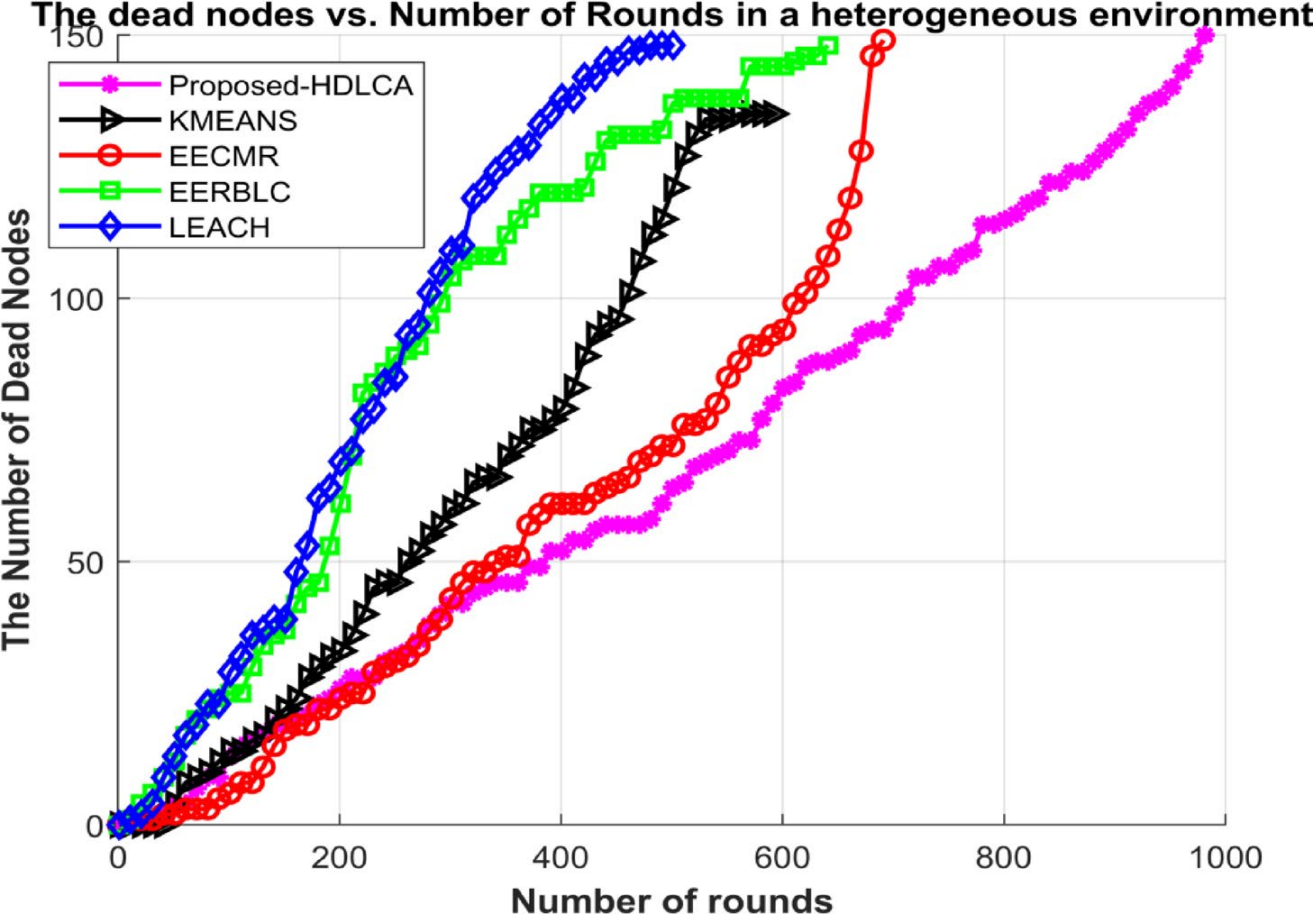
Average Residual Energy Vs. Number of Rounds in a heterogeneous environment.

**Fig. 17:**
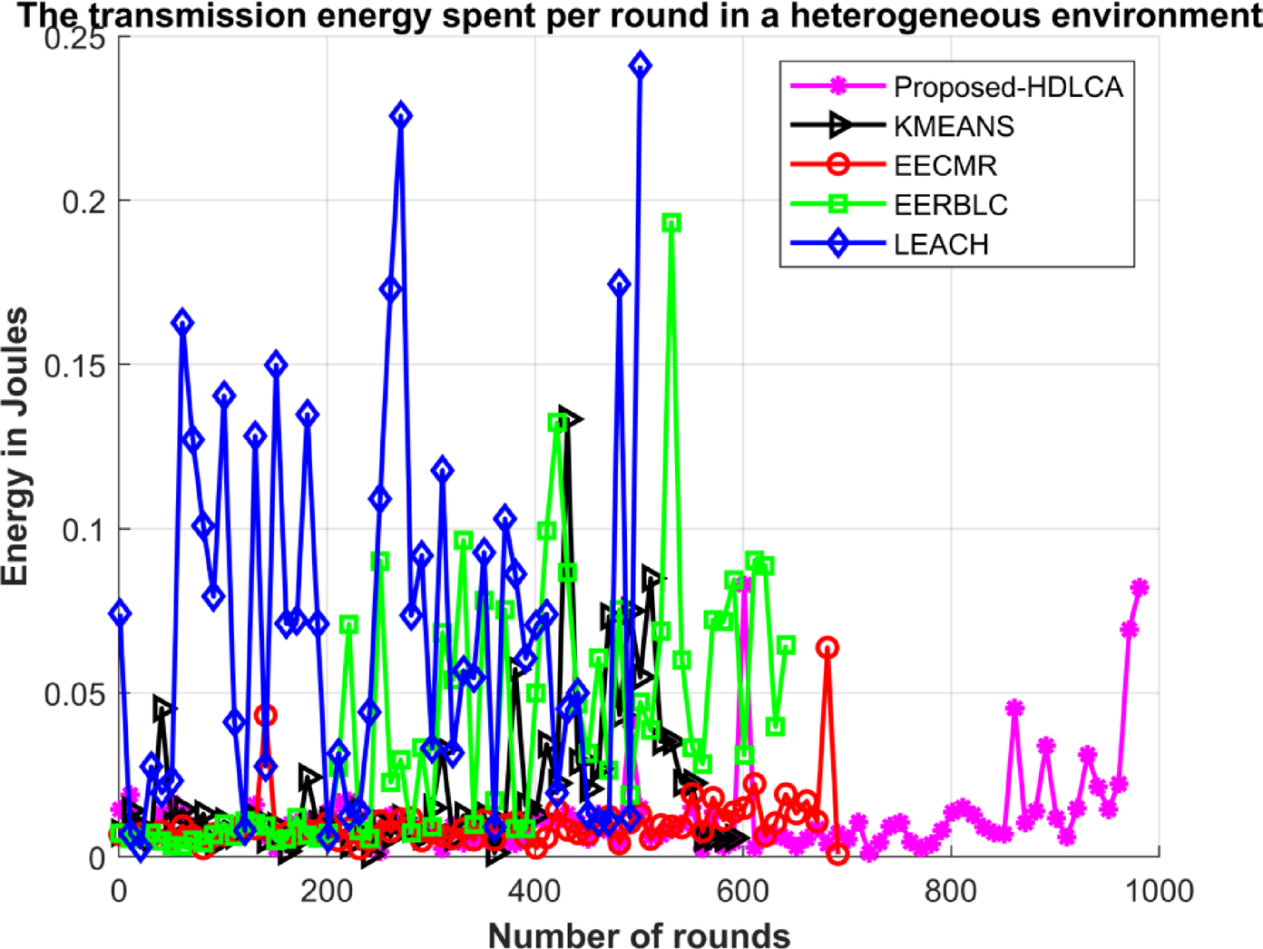
Transmission energy spent per round in a heterogeneous environment.

The sensor nodes were initially assigned random energy levels, uniformly distributed across the network. As a result, the total cumulative energy amounts to slightly more than 2.5 joules. The Fig. 15 depicts the average residual energy spent with the growing number of rounds. The curve fitting of the HDLCA clarifies that it has a slopy linear exploitation. The fact suggests that the HDLCA has a longer drive compared to all the other algorithms. The EECMR has a quick exhaust of sensor nodes and because of this the throughput of the network is affected. The EERBLC shows underperformance compared to the homogeneous environment, because unequal clustering requires to estimate the residual energy at each round. The curve of exploitation for the EERBLC has an unsteady polynomial curve suggesting an improper load balancing. The K Means clustering algorithm shows a moderate performance compared to other algorithms. The K Means clustering algorithm has an improper fitting curve. The curve of exploitation of K Means clustering algorithm reports an ineffective load balancing of the algorithm. The Fig. 17 depicts the transmission energy spent per round explains the performance of all the algorithms. The EERBLC, LEACH and the K Means clustering has a wavering response because of improper load balancing. The second most competitive algorithm EECMR spends adequate energy to reach an efficiency of 28.6% compared to the HDLCA. The load balancing property of the HDLCA influenced the transmission energy by intelligent clustering and maintaining a higher throughput.

**Table 7:** Expectancy of algorithms in heterogeneous environment.

Algorithms	Number of Rounds	First Node death	Last node death	Rate of exploitation
Proposed- HDLCA	981	19	981	Linear
EERBLC	649	3	649	Exponential
EECMR	693	2	693	Exponential
K Means Clustering	600	1	600	Higher order Exponential
LEACH	510	1	510	Intermediate

**Fig. 18:**
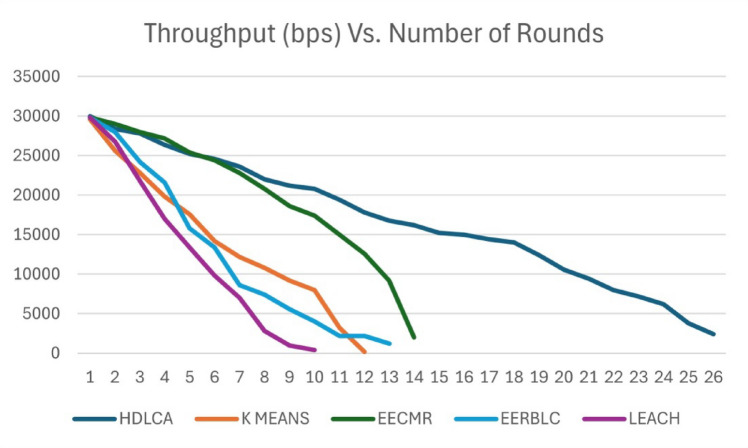
Throughput Vs. Number of rounds in a heterogeneous Environment.

The depreciated results of all the algorithms can be compared to the case of homogeneous environment. The most vital parameter to be noted is the first node death in both the cases. All the algorithms have lasted over half their lifetime delivering full throughput to the system, but the same results are not revoked under heterogeneous environment, since the energy of the system follows a normal distribution, which implies that most of the deployed nodes were initialised with a little more or less than 2.5 joules. The proposed, HDLCA shows the best result of all the algorithms. The depiction of the Fig. 16 unveils the fact that the algorithms are deviated from their behaviour compared to the homogeneous deployment. The Table 7 classifies the exploitation of these algorithms as linear, exponential, and intermediate. The deviated behaviour of EERBLC from its stepped response can be accounted for its inability to estimate the proper clustering size. The EECMR that gave a spike response in the case of homogeneous environment alters its response to be exponential. The rate of exploitation has peaked, but the steady decline curve reduces the computational load for the base station, since these nodes can be easily clustering with previous history.

The learning-based algorithm, K Means clustering shows similar response in both the situations suggesting the maximum efficiency of the algorithm has been attained. The pioneering algorithm, LEACH gave a similar response to that of homogeneous environment. It has intermediate exploitation. The proposed algorithm, HDLCA gave a phenomenal result comparatively. The linear rate of exploitation of HDLCA, suggests that the network is trained to be adjustable by sequentially modifying the parameters such as troph ic ration, hunger level, weights. The HDLCA gave a linearly declining throughput response corresponding to the number of alive nodes at any round. The HDLCA has a decelerated exploitation compared to all other algorithms. The first node death of HDLCA occurs at 19th round. The HDLCA algorithm is proven to be efficient because of the steady exhaustion of energy. The transmission energy spent per round provides the ground to support the efficiency of the algorithm. The wavering response from LEACH, EERBLC and K MEANS clustering has accelerated the exploitation of nodes. The Fig. 18 depicts the throughput of these algorithms compared at a sample of 50 rounds. The proposed HDLCA having a decline linear plot clarifying the maximum resource availability until its expectancy.

### Noise and noise free environment

Underwater wireless communication is vital for applications like submarine communication, underwater exploration, seismic monitoring and sensor networks. However, it is severely affected by various type of noises that degrades the quality of the signal and reduces the reliability of data transmission. This article aims to mitigate the impact of noise on signal transmission and establish a reliable communication link using an efficient cluster-based routing scheme. The study specifically addresses four types of noise that are inherently unavoidable in underwater environments. The considered noise sources include turbulence noise, shipping noise, wave-induced noise, and thermal noise. The Eqs. (5), (6), (*7*), (*8*) describe the power spectral densities of turbulence noise, shipping noise, wave noise and thermal noise respectively. The presence of turbulence noise is only considered under lower frequencies.

Turbulence noise arises from the random and chaotic motion of water, especially near the surface of the moving water. It typically affects the low frequency spectrum and can interfere with signal transmission particularly in shallow areas with strong currents. These turbulence noise introduces slow-varying fluctuations in the speed of sound, leading to phase and amplitude distortion. Shipping noise is one of the dominant sources of man-made underwater noise, especially in busy marine routes. The main cause of these noises are ship engines, propeller cavitation and hull vibrations. This kind of noises affects a wide range of frequency especially between 10 Hz and 1 kHz. Wave noise is a noise induced from the wave motion. These noises are produced by breaking waves, surface agitation and bubble formation. The main cause of these noises is the wind. The wave noise dominates the mid to high frequency range and varies with sea conditions. Thermal noise is caused by the random motion of water molecules at the microscopic level. It is like Johnson-Nyquist noise in electrical systems and becomes significant at frequencies above 10 kHz. While generally lower in intensity than other noise sources, it still limits the performance of high-frequency underwater communication.

**Fig. 19:**
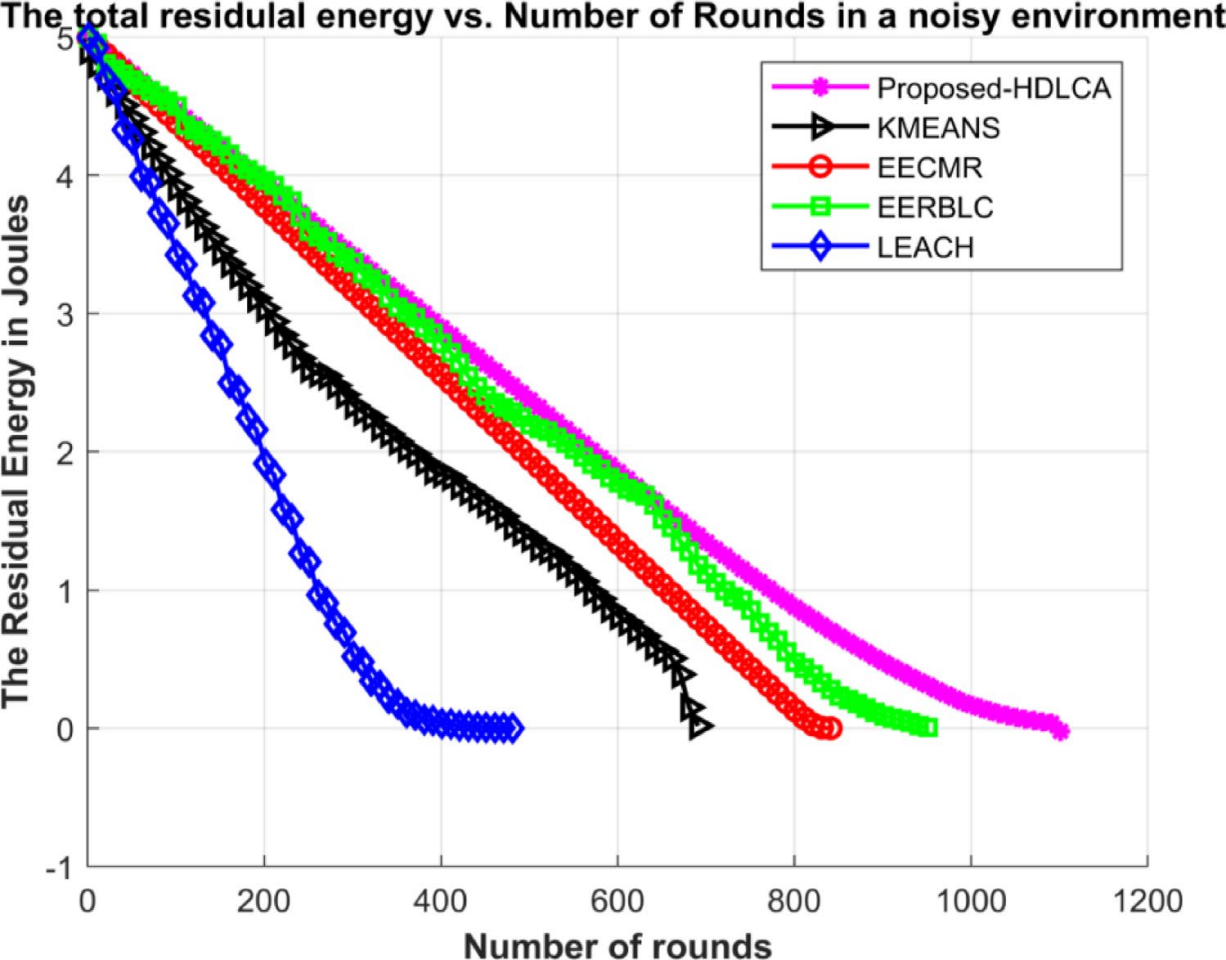
Average residual energy of the network under noisy environment.

**Fig. 20:**
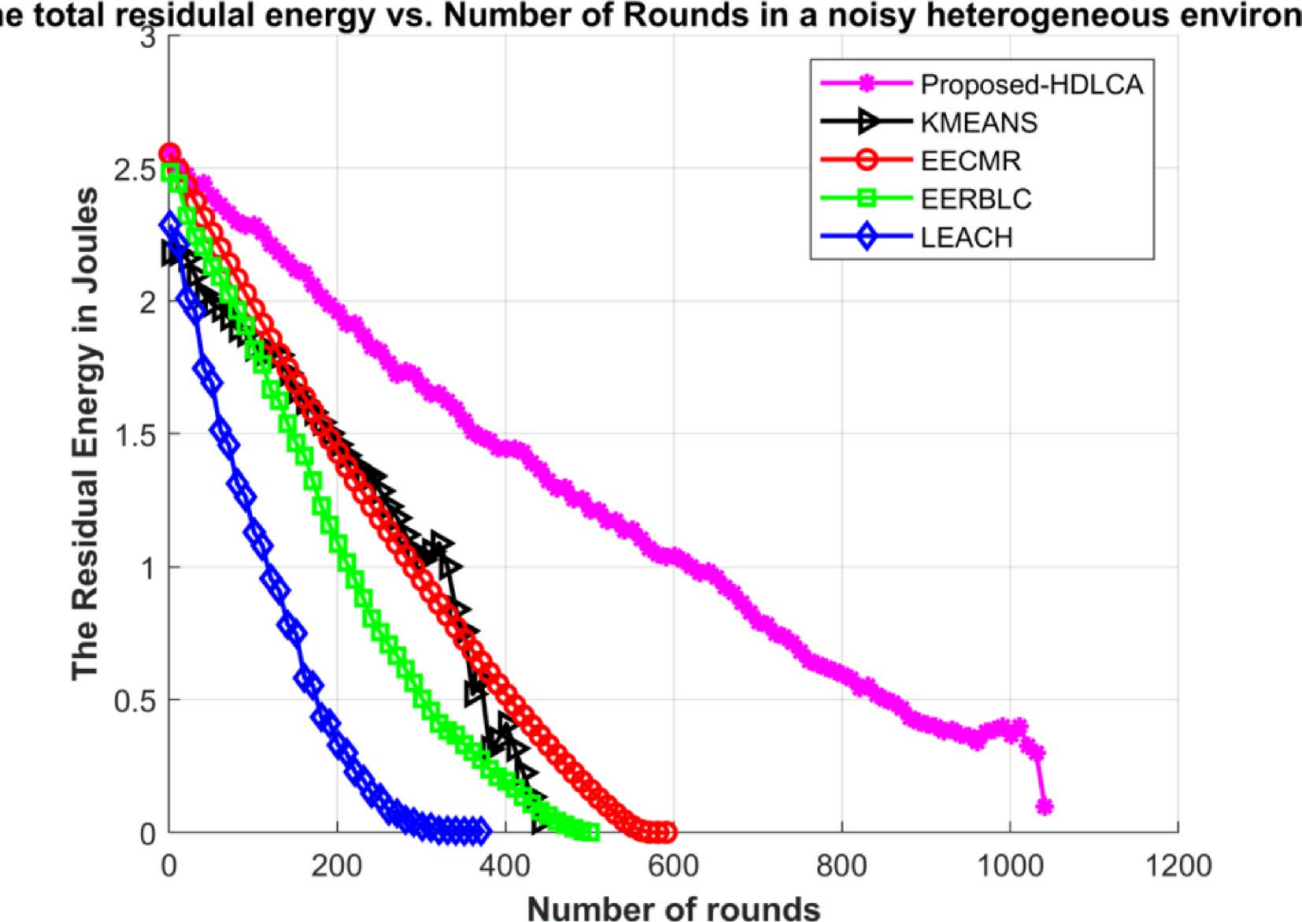
Average residual energy of the network under noisy heterogeneous environment.

**Fig. 21:**
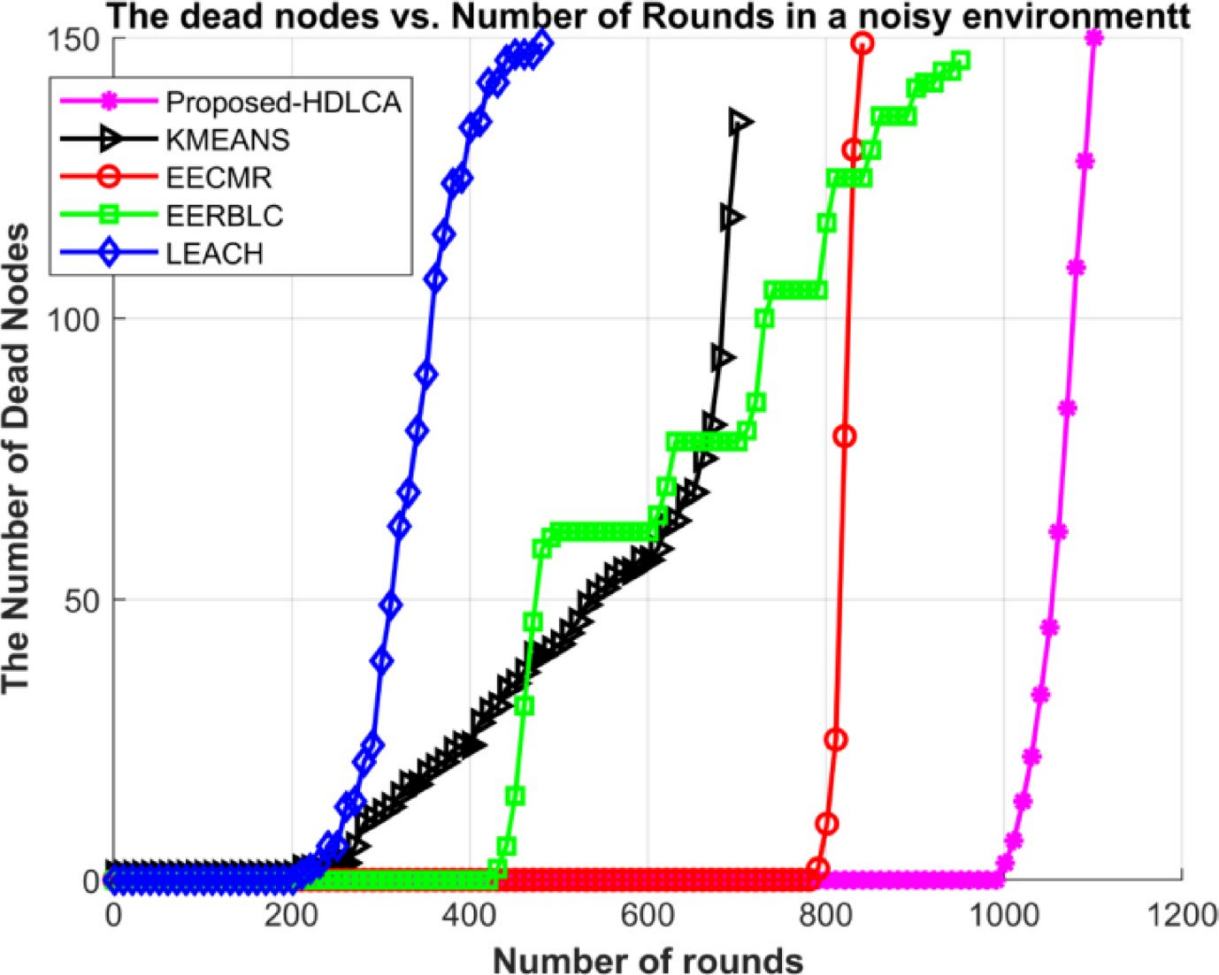
Number of dead nodes in a noisy homogeneous environment.

**Fig. 22:**
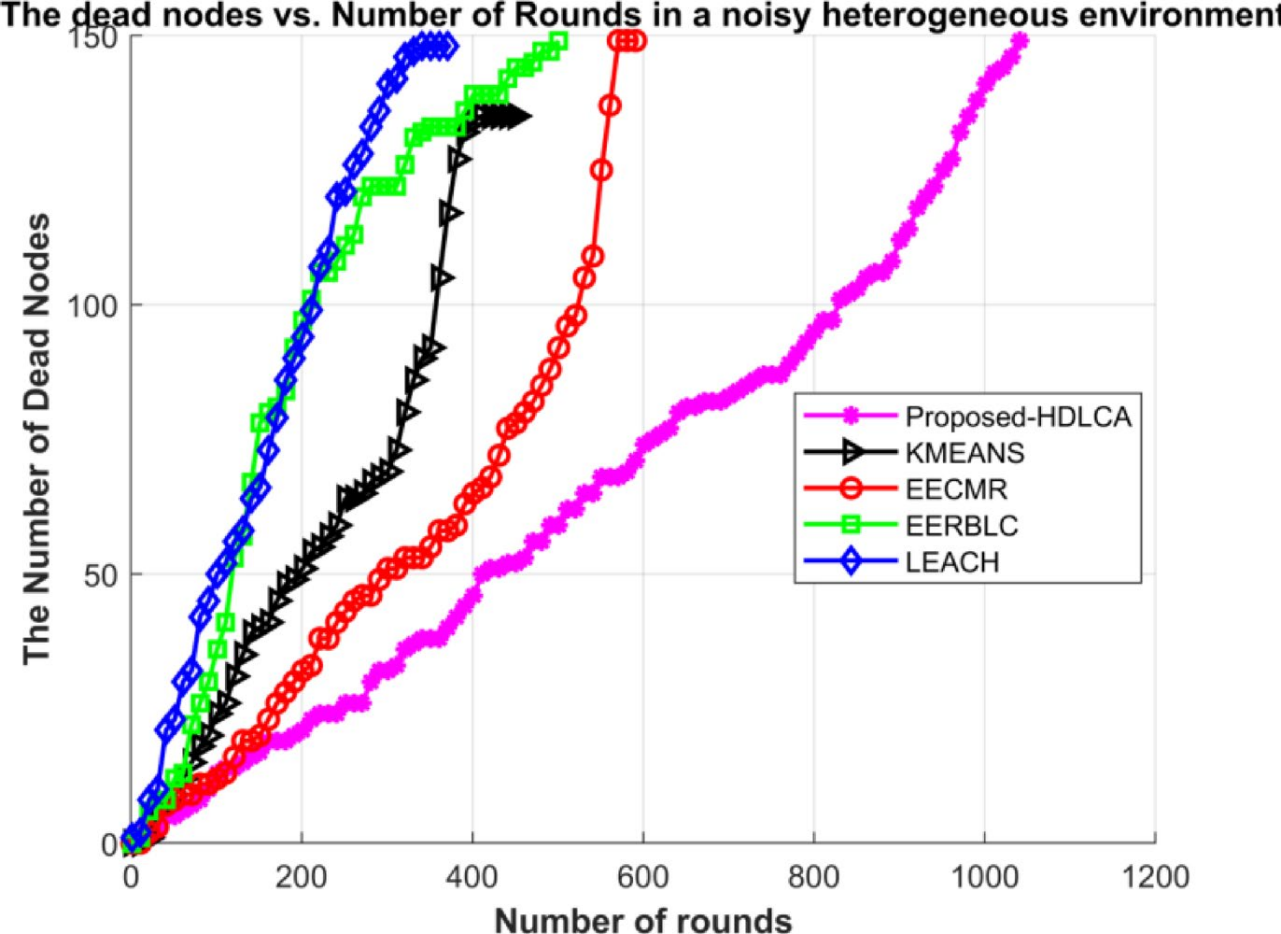
Number of dead nodes in a noisy environment.

The Figs. 19 and 20 demonstrates the average residual energy of the network in a homogeneous noisy environment and heterogeneous noisy environment respectively. The behavioural plot of the residual energy can be compared to the residual energy plot in ideal case as depicted in the Figs. 9 and 15. This suggests that the noise component is additive in nature. The additive components can shift the graph either horizontally or vertically. In the case of noisy simulation, the graph has been shifted left with similar slope. Despite of the depreciated results the proposed algorithm, HDLCA has shown phenomenal performance compared to all the other algorithms. The HDLCA lasted for up to 1101 rounds which is 256 rounds lesser than the homogeneous environment. The EERBLC algorithm has lasted up to 959 rounds which is 198 rounds lesser than the homogeneous environment. The EERBLC has a competitive efficiency but has a very low throughput for half the lifetime. The EECMR which has lasted for 846 rounds has a short of 192 rounds to the homogeneous environment. The EECMR shows better resistance to the noise compared to the foresaid algorithms. The EECMR has a deficiency of only 17.11% while HDLCA and EERBLC has a deficiency of 18.86% and 18.49%. The K Means clustering has a deficiency of 20.67% which is the highest among all the algorithms. The LEACH has the least deficiency of 10.84% and quantitively short of 59 rounds only compared to the homogeneous environment.

**Table 8:** Performance metrics of the algorithm under ideal case and noisy case.

Algorithm	Number of rounds in ideal homogeneous environment	Number of rounds in noisy homogeneous environment	Number of rounds in ideal heterogeneous environment	Number of rounds in noisy heterogeneous environment
Proposed- HDLCA	1357	1101	981	1047
EECMR	1038	846	693	598
EERBLC	1157	959	649	506
K MEANS	895	710	600	456
LEACH	544	485	510	378

The Table 8 depicts the performance metrics of all the algorithms in terms of number of rounds. The obtained results shows that the proposed algorithm performs well under very deployment. The best-case scenario is homogeneous ideal deployment, which returns a maximum efficiency for every algorithm but with higher level of mitigation the performance degrades exponentially. The Figs. 21 and 22 provides the visual depiction of the nodal expectancy of the competitive algorithms. The worst-case scenario which is heterogeneous noisy deployment has the lowest return of efficiency because of higher level attenuation. The proposed HDLCA, has a degradation of 22.84% while every other algorithm has a degradation of over 30%. This ability of higher noise resistance proves to be competitive to maintain the throughout and the reliability of the network.

### Role of nomad nodes

**Fig. 23:**
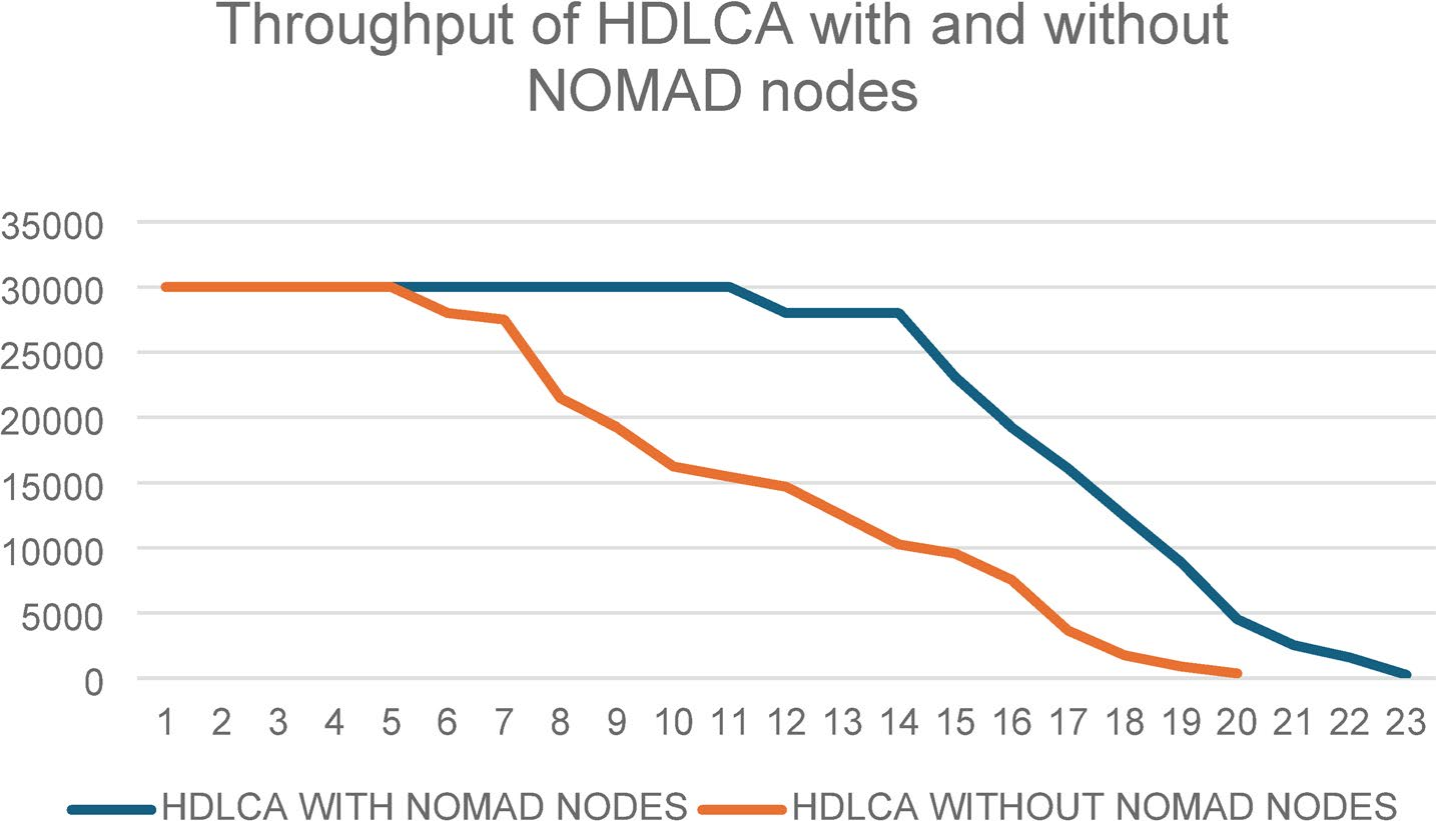
Comparison of throughput in HDLCA with and without nomad nodes over successive transmission rounds.

The inclusion of nomad nodes in HDLCA significantly influences the overall data transmission performance. As shown in Fig. 23, the throughput of HDLCA with nomad nodes consistently outperforms the configuration without them across all rounds. This improvement is attributed to the ability of nomad nodes to dynamically assist in routing, thereby reducing packet loss and enhancing delivery efficiency.

Figure 23 illustrates the comparative throughput performance of HDLCA with and without nomad node integration. It is evident that the inclusion of nomad nodes significantly enhances network throughput, particularly in the later rounds. While both versions maintain similar performance during initial rounds, the variant incorporating nomad nodes sustains higher throughput for a prolonged duration. This validates the role of nomad nodes in load balancing and route reinforcement, effectively preventing premature node failures and reducing packet loss. The result confirms that dynamic involvement of nomad nodes enhances overall network robustness and data delivery efficiency.

### Computational complexity

The computational complexity of clustering algorithms plays a crucial role in determining their feasibility for real-time underwater sensor networks where energy and processing power are limited. The computational load of clustering algorithms is vital to ensure energy-efficient and real-time operation in such challenging environments.

**Table 9:** Computation metrics of the algorithms.

Algorithms	Elapsed time	Time Complexity	Key factors
Proposed- HDLCA	0.136517 s	O (n^2^)	Iterative behaviour
EECMR	0.1058 s	O (n log n)	Energy aware sorting
EERBLC	0.0950002 s	O (n log n)	Layered structure
K MEANS	0.06763 s	O (n)	Centroid updates
LEACH	0.060864 s	O (n)	Random head selection

The elapsed time has been calculated using the timer in the MATLAB version 2024b. The Table 9 depicts a computational study of different algorithms compared with the proposed algorithm to test the complexity. The proposed algorithm being a bio inspired algorithm and behaviour driven, involves iterative updates of the hunger level and calculation of depth for routing. This leads to a complexity of O(n^2^), where n denotes the number of sensor nodes. The energy aware clustering algorithm such as EECMR and EERBLC have intense calculations of residual energy and distance whose complexity is O (n log n) due to sorting and cluster head selection mechanism. K-Means, a classical clustering method, has a complexity of O(n), where n is the number of nodes and making it computationally efficient but less adaptive to dynamic network conditions. LEACH has the lowest complexity among all, approximately O(n), as it uses probabilistic cluster-head selection and periodic re-clustering without intensive optimization. However, this simplicity comes at the cost of reduced stability and lower network lifetime. While HDLCA demands high computational resources, it compensates with superior resilience, adaptability and energy balance.

## Conclusion

In this study, we propose an efficient and scalable clustered routing model aimed at reducing energy consumption and enabling seamless scalability from small to large network areas. Moreover, a novel cluster-based routing protocol named HDLCA has been developed, inspired by the trophic behaviour of lions. The proposed methodology has proven to be energy-efficient and has significantly increased the network lifetime. The newly formulated fitness function, integrated with the proposed hunger mechanism, has greatly improved the throughput and scalability of the network. Through comparative analysis, it was found that HDLCA utilizes network energy more efficiently than several existing clustering protocols. In addition to significantly improving throughput, HDLCA has proven to be robust against heterogeneous deployments and noisy environments.

Our future research focuses on reducing computational load through behavioural modelling and the integration of a machine learning model that defines appropriate parameters and updates them at regular time intervals. Additionally, we aim to develop a hybrid collaboration model, which is expected to deliver faster convergence, thereby improving throughput, latency, and computational efficiency. Furthermore, our future work extends toward enabling secure and reliable underwater communications. Since routing is highly vulnerable in UWSNs, employing secure or location-based clustering can significantly enhance the network’s security. With this objective in mind, we plan to study lightweight encryption and MAC-based integrity mechanisms to ensure secure data transmission. In parallel, we will further work on implementing the algorithm in firmware to validate the performance and its implementation in practical life. This work lays the foundation for further innovations in wireless communication and bio-inspired optimization, ultimately advancing the practical deployment of intelligent sensing systems.

## Data Availability

The data that support the findings of this research are available on request from the corresponding author.
